# How and why humans trust: A meta-analysis and elaborated model

**DOI:** 10.3389/fpsyg.2023.1081086

**Published:** 2023-03-27

**Authors:** P. A. Hancock, Theresa T. Kessler, Alexandra D. Kaplan, Kimberly Stowers, J. Christopher Brill, Deborah R. Billings, Kristin E. Schaefer, James L. Szalma

**Affiliations:** ^1^Department of Psychology and Institute for Simulation and Training, University of Central Florida, Orlando, FL, United States; ^2^Department of Psychology, University of Central Florida, Orlando, FL, United States; ^3^Department of Management, University of Alabama, Tuscaloosa, AL, United States; ^4^United States Air Force Research Laboratory, Wright Patterson Air Force Base, Dayton, NV, United States; ^5^Broky Consulting, LLC, Hillsboro, OR, United States; ^6^DEVCOM Army Research Laboratory, Aberdeen Proving Ground, Adelphi, MD, United States

**Keywords:** trustors, trustees, meta-analysis, trust, dispositional trust

## Abstract

Trust exerts an impact on essentially all forms of social relationships. It affects individuals in deciding whether and how they will or will not interact with other people. Equally, trust also influences the stance of entire nations in their mutual dealings. In consequence, understanding the factors that influence the decision to trust, or not to trust, is crucial to the full spectrum of social dealings. Here, we report the most comprehensive extant meta-analysis of experimental findings relating to such human-to-human trust. Our analysis provides a quantitative evaluation of the factors that influence interpersonal trust, the initial propensity to trust, as well as an assessment of the general trusting of others. Over 2,000 relevant studies were initially identified for potential inclusion in the meta-analysis. Of these, (*n* = 338) passed all screening criteria and provided therefrom a total of (*n* = 2,185) effect sizes for analysis. The identified dependent variables were trustworthiness, propensity to trust, general trust, and the trust that supervisors and subordinates express in each other. Correlational results demonstrated that a large range of trustor, trustee, and shared, contextual factors impact each of trustworthiness, the propensity to trust, and trust within working relationships. The emphasis in the present work on contextual factors being one of several trust dimensions herein originated. Experimental results established that the reputation of the trustee and the shared closeness of trustor and trustee were the most predictive factors of trustworthiness outcome. From these collective findings, we propose an elaborated, overarching descriptive theory of trust in which special note is taken of the theory’s application to the growing human need to trust in non-human entities. The latter include diverse forms of automation, robots, artificially intelligent entities, as well as specific implementations such as driverless vehicles to name but a few. Future directions as to the momentary dynamics of trust development, its sustenance and its dissipation are also evaluated.

## Introduction

Trust is one of the principal forces which binds society together (Hobbes, 1651; Redfern, 2009). All forms of social interaction involve at least some degree of implicit or explicit trust, singly or in conjunction. Trust is, necessarily, an emergent property. This is because it derives from this interaction between at least two or more entities; one of whom trusts and another who is trusted. Trust can be, and often is, reciprocal. Trust itself is expressed by the information conveyed *via* a communication channel linking all of these persons and/or other responsive entities together (see, e.g., Shannon and Weaver, 1949; Wiener, 1950). Phenomenologically, trust represents the affective-cognitive states of those involved in these respective relationships. Trust is therefore an integral facet of conscious experience for both the trustor (the person that trusts) and the trustee (the one who is to be trusted). Whether and how such affect can be experienced by other animals and now more especially non-living computational entities is the subject of much current debate (e.g., Phillips et al., 2016; Maehigashi et al., 2022). Non-living systems here can include the set of nascent autonomous agents that are rapidly entering our everyday lives (see, e.g., Hancock et al., 2019; Splegelhalter, 2020; Kaplan et al., 2021). The similarities and differences with which trust is placed in these differing entities is also now much in discussion (Madhavan and Wiegmann, 2007). In previous work, we have indicated that trust can be defined as: “*an individual’s calculated exposure to the risk of harm from the actions of an influential other*” (Hancock et al., 2011a). Relatedly here, we take risk to be the potential for, and thus probability of, harm or injury. In its turn, harm represents the degree of physical and/or psychological damage that can occur to the trustee (or group of trusting individuals or entities) resulting from any incorrectly calibrated trust decisions (and see Hancock et al., 2020). This description serves to encapsulate the wide variety of definitions found in the varying fields of literature addressed here. It therefore allows for aggregation of the term trust across differing studies and multiple domains. Of course, trust also necessarily exhibits positive value, and its persistence indicates that, as a general proposition, the social value of trust outweighs its potential downsides, on average. Indeed, global responses to evident threats such as the recent pandemic are predicated upon these collective social trust response (Bollyky et al., 2022). As to whether and how these traded values are expressed in singular interactions, the following work is directed to explore.

### Modeling trust development

**Figure 1:**
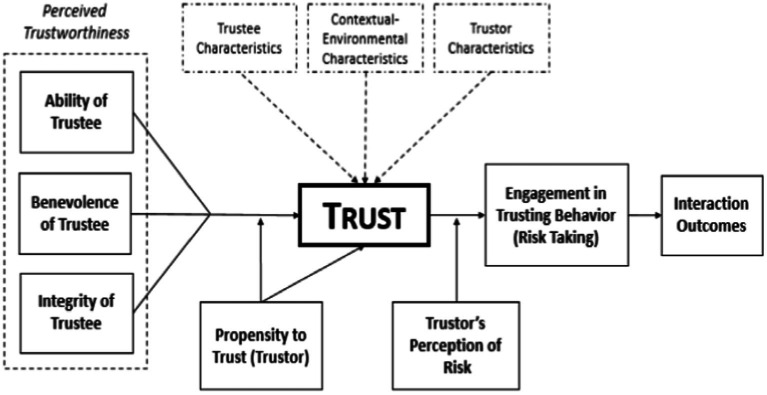
The model of interpersonal trust originally proposed by Mayer et al. (1995). This original trust model includes trustworthiness (a trustee characteristic), the propensity to trust (a trustor characteristic), and interactive outcomes (one element of the contextual/environmental factor) as the primary elements that impact trust development and maintenance. We argue this model is limited in scope and that, for a full exposition, additional trustee, and trustor characteristics as well as extensive contextual factors need to be considered in human interpersonal trust development. These are here represented by the dashed boxes at the top of the illustration.

**Figure 2:**
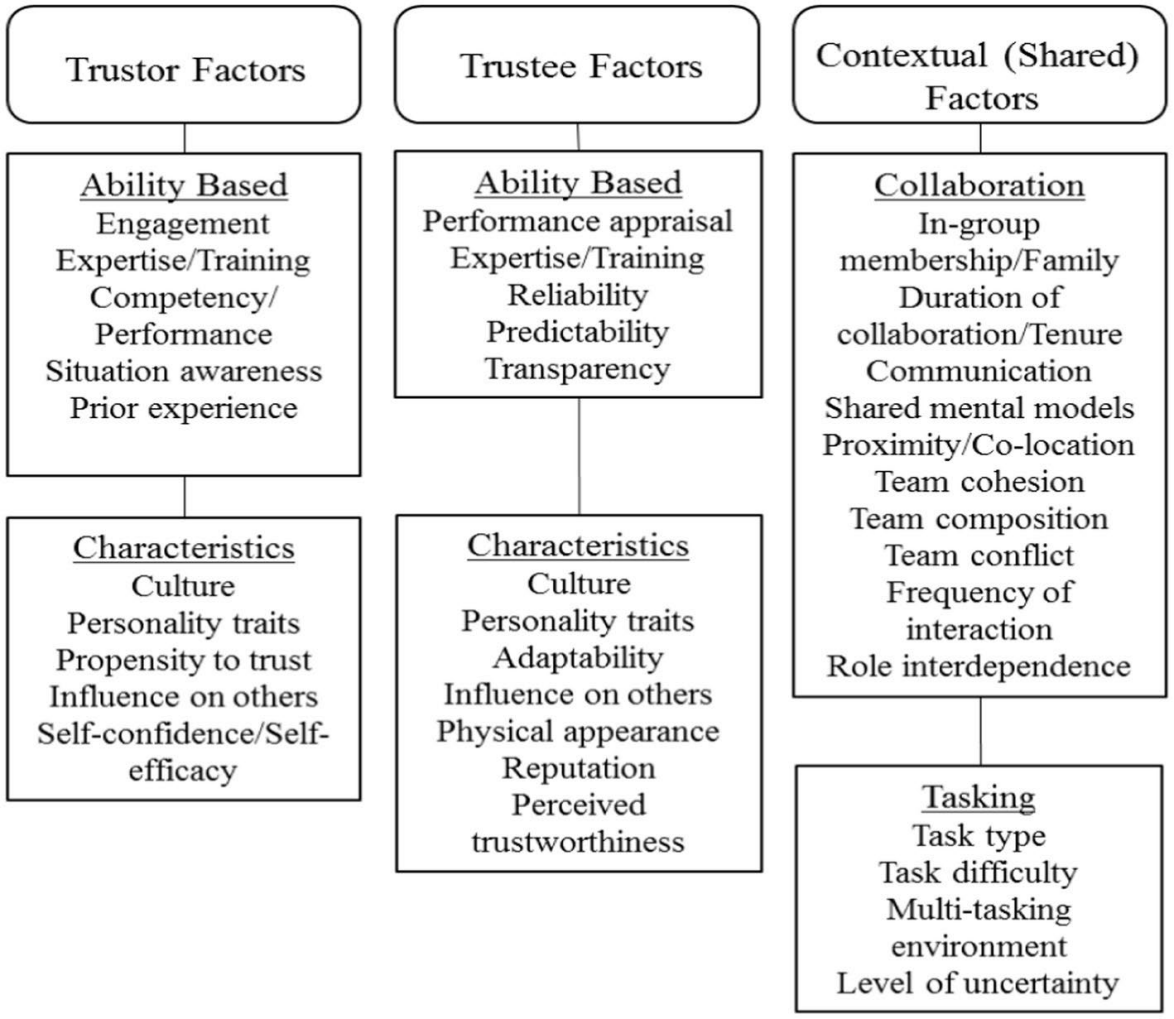
Factors identified as potential antecedents of interpersonal trust prior to any coding of the collected empirical studies.

Figure 1 illustrates our basic model of trust and its development. This model is founded upon the prior work of Mayer et al. (1995). In the present version, we have superimposed a triad of factors which are explicitly derived from our own program of experimental and integrative work (see, e.g., Hancock et al., 2011a, 2021a,b; Kaplan et al., 2020). In particular, we emphasize a dimension not previously integrated with extant human trust research, namely the context within which the trust interaction occurs. Further, from our survey of extant work, we have identified a number of other influences which have received sufficient attention to support their impact on human trust situations, the degree to which those effects are significant being the subject of quantitative and qualitative assessment here. We suggest that this elaborated model provides the most useful framework around which to begin to explore and integrate the empirical pith that our present meta-analytic investigation illuminates. In alignment with the model’s structure, experimental studies from each of the peer-reviewed works that were included in the present meta-analysis, were classified into our three overarching categories. These being (1) trustor factors (i.e., factors associated with the characteristics of the individual who trusts), (2) trustee factors (i.e., factors related to the characteristics of the individual in whom trust is placed), and (3) contextual factors (i.e., situational and environmental factors shared between the trustor and trustee at the time of their interaction). These categorizations enable a principled, quantitative review of the predictive strength of each of the respective identified factors in human interpersonal trust. Antecedents of trust were also identified from each of the contributory studies and included in coding the assemblage of statistical data. Figure 2 provides a complete listing of these factors which were identified as potential influences on interpersonal trust based on our current review of the panoply of extant literature that we surveyed.

### Factors associated with the trustor

Previously, we (Kessler et al., 2017) have identified a range of trustor factors that include both ability-based characteristics (e.g., expertise, competency, degree of prior trust experience, etc.) in tandem with individual characteristics (e.g., demographic profile, personality traits, and attitudes toward others), which mediate trust interactions. For example, research suggested that personal factors such as reputation, propensity to trust, and gender each influence trust development (see also, Johnson-George and Swap, 1982; Axelrod, 1984; Irwin et al., 2015). Further, in their model of interpersonal trust, Mayer et al. (1995) asserted, quite logically, that the trustor’s propensity to trust is a large and perhaps the most influential factor in trust development. Also, emotional state has been found to be associated with interpersonal trust in a series of empirical studies (see, e.g., Dunn and Schweitzer, 2005; Myers and Tingley, 2016). Further, a trustor’s cognitive abilities and personality (i.e., agreeableness and openness) are also linked to trust in interpersonal relations. This is most particularly evident in distributed teams (Adams and Webb, 2002; Stokes et al., 2011). In consideration of these and like observations, we anticipated that the factors relating to the trustor’s personal characteristics and abilities (as specified in Figure 2) would play substantial roles in the development of trust in interpersonal relationships.

### Factors associated with the trustee

Identified and influential trustee factors have included both performance-based characteristics (e.g., behavior, predictability, and reliability) as well as individual characteristics (e.g., personality, reputation, etc.; see Kessler et al., 2017). The predictability of, and expectations of, a trustee are each key elements of trust (Bhattacharya et al., 1998). Research has also suggested that a trustee’s perceived ability, their benevolence, and integrity (i.e., elements which largely then comprise the trustee’s trustworthiness) are most associated with trust in interpersonal relationships (Mayer et al., 1995; Amogbokpa, 2010). The abilities exhibited by a trustee are also especially taken into consideration and are predominant influences as trust develops. For example, in two studies, people perceived to be higher in ability were trusted more than those perceived of as low in that same ability (Ammeter, 2000; Poon, 2013). The different characteristics of the trustee that are perceived by the trustor thus impact the levels of trust involved in the interaction (and see also Ridings et al., 2002).

### Factors associated with the context of trusting

Finally, the contextual factors that our research groups have identified as potentially impactful on trust include both collaborative characteristics (e.g., culture, communication, and shared mental models) as well as task characteristics (e.g., work environment, level of uncertainty, and task complexity; and see Kessler et al., 2017). Working with any other person or entity to accomplish a common task requires inter-dependence, at least to a degree, in order to achieve such mutual goals (Mayer et al., 1995). The degree of this interdependence has been identified as one of the most important factors involved in the development of trust (Axelrod, 1984). Other critical environmental antecedents have also been established in the existing literature. For example, human-human communication factors contribute to trust relationships (Axelrod, 1984), as do acceptance of responsibility, confidentiality, integrity, consistency in behavior, and emotions, as depicted through non-verbal communication channels. All represent key themes in trust development (Akinyele, 2004). Social identification with in-groups, as well as dissonance with out-groups, can further contribute to biases in perceptions of trustworthiness and these factors frequently serve to guide the trust in others (Ammeter, 2000). Racial and ethnic similarities have been found to be associated with baseline affect-based trust, where co-workers of a similar race and ethnicity initially trust each other more (Ammeter, 2000). However, this propensity was not found to be as strong a determinant after the co-workers had interacted over a more extended periods of time. This is one of a plethora of indications that trust is a constantly developing and evolving aspect of conscious interactive experience and argues for a greater focus on the dynamics of trust in future research endeavors.

Trust can be most especially important in situations where there is no direct face-to-face interaction. This occurs when team members communicate remotely *via* different forms of media (Bandow, 1998) and has been an especial feature of our modern “zoom” era. Such influences of social and electronic “distance” on trust has therefore been most particularly relevant during the recent pandemic isolation situation (e.g., Kimmel, 2020). Increased levels of trust in these various contexts has been found to promote more positive work group experiences among distributed teams of individuals. However, support has also been found for the notion that higher absolute levels of trust reside in local team members and proximal partners, as compared to others who are then classed as more “remote.” This is the case even though the latter engage in frequent interactions (Aubert and Kelsey, 2003). These, and other shared factors (as specified in Figure 2) were each identified as potentially influential in the development of, and sustenance of interpersonal trust. Along with each of the aforementioned trustor and trustee factors, these founding, contextual premises helped shape and guide the structure of our meta-analytic evaluation, the detailed specifications of which now follow.

## The present evaluation method

The examination of trust across diverse disciplines carries with it certain limitations. Most notably these include the fact that trust is measured and operationalized differently in its various operational contexts. However, as has been readily acknowledged (Rousseau, 1998; Dirks and Ferrin, 2002) even differing definitions of trust do share some evident, common core concepts. Most prominently, this features the dimension of exposure and thus the notion of trust as a willingness to be vulnerable to another party. It is upon this commonality that our meta-analysis is grounded. By starting from this ubiquitous origin of risk and reward, we are able to explore the widest range of relevant works, while also pinpointing areas for needed further exploration. The fundamental question across all of the surveyed domains proves to be a relatively simple one: how is human trust developed and maintained? And as a corollary, what causes it to then be maintained and/or subsequently dissipate, dissolve, and disappear? To answer these questions, we first accessed all of the available, extant meta-analyses that have previously been conducted and reported on interpersonal trust. These works proved to be informative but were, however, often limited in their scope. Thus, each individual report was confined to research upon one particular aspect of trust. In consequence, these respective reports did not, individually, assess the entirety of the interpersonal trust domain. For example, Colquitt et al. (2007) assessed only relationships between trust, trustworthiness, trust propensity, and the behavioral outcomes of such interactions, such as risk-taking and job performance. Likewise, Dirks and Ferrin (2002) focused their meta-analysis on the relationships between trust in leadership, trust antecedents, and the interactive outcomes of these two issues. Yet another meta-analysis reported by Maguin (2010) examined only the associations between interpersonal trust and various team performance characteristics. And most recently, two of our own trust meta-analyses have focused on concerns largely beyond the human-human realm by featuring human-robot trust (Hancock et al., 2011b, 2021a,b). Yet another such work has reported on the broader aspects of human-automation trust (Hancock, 2014; Schaefer et al., 2016), while the most recent of our group’s efforts have evaluated trust in AI systems (Kaplan et al., 2021). All of these works provide relevant information and insight yet, currently, there is no broad meta-analysis which deals with the full and comprehensive body of human-human interpersonal trust research across multiple disciplinary perspectives. Therefore, the explicit purpose of our current work is to collect and quantitatively evaluate the most complete set of possible empirical studies on interpersonal trust and to analyze such data through meta-analytic procedures. This process was undertaken in order to assess the strength of identified factors impacting trust development and trust sustenance in all reported instances of human-human interaction. Additionally, these present findings serve as a basis to provide the structure of an advanced model of the operations of trust that we develop below. We begin this account with our information search procedures.

### Information search strategies

An initial literature search of peer-reviewed journal articles with no restrictions on publication date was conducted using a multiplicity of library databases. These databases spanned differing disciplines, including, but not limited to, *JSTOR®*, *ProQuest®*, *and EBSCOhost®*. In addition, we used web-based search engines (e.g., *Google*® and its derivative *Google Scholar*®) to identify further references not discovered by the initial, formal scan. The Boolean search strategy included 26 combinations of terms derived from our extended human-human trust model as well as all of our previous meta-analyses and those of others as referenced above. All of these terms and their combinations are specified in Table 1. Terms encompassed in other identified search terms were excised. Also, the trustee characteristic of “adaptability” was not directly included. This is because adaptability represents a performance measure and was, as a result, included in the identified search term for performance effects. Additionally, the contextual factors, “task type” and “environment,” were not included in the initial scan. This is because task and environment cannot be either controlled or randomly assigned in this field of concern. These latter terms were thus excluded from the beginning search profile but were reserved for, and still included in subsequent analysis as predictors. After an initial listing of articles was obtained, the reference list of each of these identified articles was then surveyed to determine whether any other related studies could be identified. When this latter elicitation process no longer yielded any new citations, we compiled a final listing of articles as qualifying from our first screening. A total of 5,048 documents and reports were identified based on the specified search terms (Table 1).

**Table 1:** Human-human trust Boolean search terms, derived from our proposed hybrid model of human-interpersonal trust development.

Human-interpersonal trust literature Boolean search terms	
1. “Engagement”	14. “Group composition”
2. “Performance”	15. “Uncertainty”
3. “Situation awareness”	16. “Self-efficacy” OR “self-confidence”
4. “Prior experience”	17. “Expertise” OR “expert”
5. “Culture”	18. “Reliability” OR “predictability”
6. “Propensity to trust”	19. “Physical appearance” OR “attractiveness”
7. “Transparency”	20. “In-group membership” OR “group identification”
8. “Reputation”	21. “Mental models” OR “mental model”
9. “Trustworthiness”	22. “Conflict” AND (“team” OR “organization” OR “group”)
10. “Tenure”	23. (“Interaction frequency” OR “frequency of interaction”)
11. “Communication”	24. (“Task interdependence” OR “role interdependence”)
12. “Proximity”	25. (“Task difficulty” OR “multitasking”)
13. “Cohesion”	26. “Team composition” (OR “organizational composition” OR “group composition”)

All search terms (or combinations of search terms) were paired with: (“trust” AND).

### Inclusion and exclusion criteria

After removing duplicates and works that were not peer-reviewed or were not obviously reflective of some evident facet of human-human trust, a total of 1,909 documents remained. These were then further inspected to ensure that they passed the following criteria for inclusion in our meta-analysis. Specifically:

1. Each document had to have reported an empirical examination (correlational or experimental) of trust in which trust was a measured outcome.
2. Study participants had to consist of persons over 18 years of age and not include individuals who were otherwise vulnerable or represented clinical populations.
3. The empirical examination of trust had to be directed toward an individual.
4. Each study had to include sufficient information to determine effect size estimates. Sufficient information was considered to consist of the population size for the study, population size by condition, means, and standard deviations by condition, and *F* tests or *t* tests. For correlational studies, *r* values were acceptable.
5. Each document must have originated from a peer-reviewed journal, dissertation, or conference proceedings.
6. There were no geographical or temporal limitations as to which papers could be included. However, all papers had to be written in English or had to have a sufficient English translation readily available.

**Figure 3:**
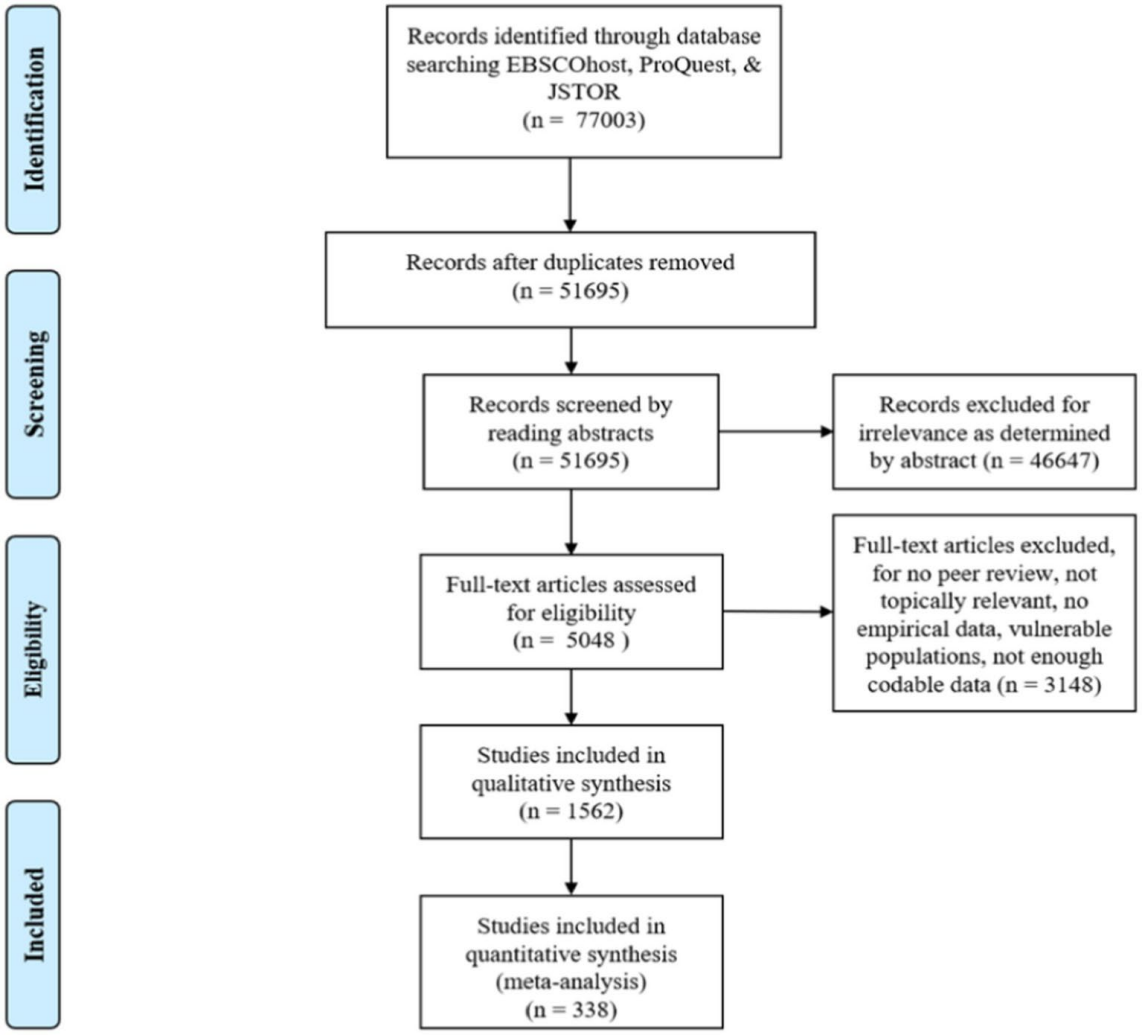
PRISMA flow diagram illustrates the sequence of study identification and sequential refinement of the presently qualifying studies.

The application of this assessment process resulted in 338 empirical articles, reports, dissertations, and conference proceedings published between 1974 and 2018 that met all of the criteria for inclusion in the present meta-analysis (see Figure 3). These 338 works reported 2,125 correlational effect sizes and 60 experimental effect sizes. When one study reported multiple effect sizes for the same pair of variables (i.e., Tenure and Perceived Trustworthiness), such results were averaged within the study in order that each sample population only contributed one final effect size for each analysis. This was done to meet the assumption of independence criterion (see Hunter and Schmidt, 2004). The 338 documents which met all the inclusion criteria in our meta-analysis are identified in Appendix A. In all meta-analyses, it is important to exclude certain identified works in order to ensure that each empirical finding is only included once. This caution is required because some data sets are published on multiple occasions, and it is important that they do not then exert disproportionate effects due to repeated inclusion. Therefore, we ensured that each set of data was represented only once in our final coded documents.

### Coding procedures and effect size calculations

The initial coding of those articles identified early in the process was accomplished by two of the present authors (DB, KS). Subsequently, the full coding of the whole panoply of identified, qualifying works was then conducted by another two of the current authors (TK, AK). These individuals undertook the coding process by dividing the full article list in two parts, and then each coded their respective half. Once that initial coding had been completed, the individuals then exchanged lists and proceeded to double-code the other’s list. As a consequence, all studies were checked at least twice and the large majority of them upon four occasions. All points of disagreement were then discussed until a consensus resolution was reached. Meta-analysis was used to evaluate the data so collected in order to determine the patterns of findings in the identified body of human interpersonal trust research. First, each study’s effect size was calculated using standard formulae (see Hedges and Olkin, 1985; Morris and DeShon, 2002; Hunter and Schmidt, 2004). As the construct of “trust” was measured in a variety of methods (e.g., different scales and behavioral measures), a random-effects model was used in calculating the present results to account for such variation (and see Hunter and Schmidt, 2004). Studies included in effect size calculations contained both correlational and group design data. Therefore, the use of multiple meta-analytic methods (correlation and Cohen’s *d*) was deemed relevant and necessary. The correlational effects represent an association between trust and any given factor. In the present work, these antecedents of trust each appear in the proposed human interpersonal trust model, as illustrated in Figure 1. Cohen’s *d* represents the standard difference between two means in SD units.

From these collective observations, we garnered correlational and causal inferences between trust and any given factor. In some cases, only one qualifying article, that examined a specific variable pair, met the inclusion criteria (i.e., *k* = 1). In these, relatively rare cases, the findings were still included in order to provide a comprehensive overview of the current state of the literature. However, in these cases (*k* = 1), confidence intervals could not be established for that effect size. Such cases cannot yet be considered truly “meta” analytic since they provide only one, idiographic outcome. However, these cases do suggest areas in the overall fabric of research in which further empirical attack is advised. In both types of the meta-analytic effects which are reported below, a positive number represents higher trust and vice versa. Findings were interpreted using established ranges for d of Cohen (1988). Respectively, these are small (*d* ≤ 0.20; *r* ≤ 0.10), medium (*d* = 0.50; *r =* 0.25), and large (*d* ≥ 0.80; *r* ≥ 0.40) effect sizes.

### Variance estimates

For all included studies, several variance estimates were calculated. First, the variability of the effect sizes themselves (*s^2^_g_*) and then variability due to sampling error (*s^2^_e_*) were estimated. Next, these two values were used to compute residual variance (*s^2^_δ_*). A final check for homogeneity of variance $\left(\frac{s_{e}^{2}}{s_{g}^{2}}\right)$ was calculated (proportion of total variance accounted for by sampling error). Hunter and Schmidt (2004) have suggested that an outcome here of 0.75 or greater suggests that the remaining variance is due to a variable that could not be controlled for and represents homogeneity of variance. However, large residual variance and large homogeneity of variance may be seen as a result of a small number of sample studies and this propensity is evident in some of our own present results. Lipsey and Wilson (2001) have provided an in-depth examination of the various strengths and weaknesses relating to these elements of meta-analytic procedures. In some cases, this process can result in a proportion of observed variance due to sampling error that exceeds unity. This can occur because the sampling error variance and the total observed variance are computed separately, and if the observed variance proves to be very small and/or the sampling error variance is then very large, the proportion can be greater than a value of 1. Such cases can be interpreted to mean that either the variances are based on only a few effect sizes (e.g., *k* = 2 or *k* = 3), or that the effect sizes are homogeneous.

### Publication bias

**Figure 4:**
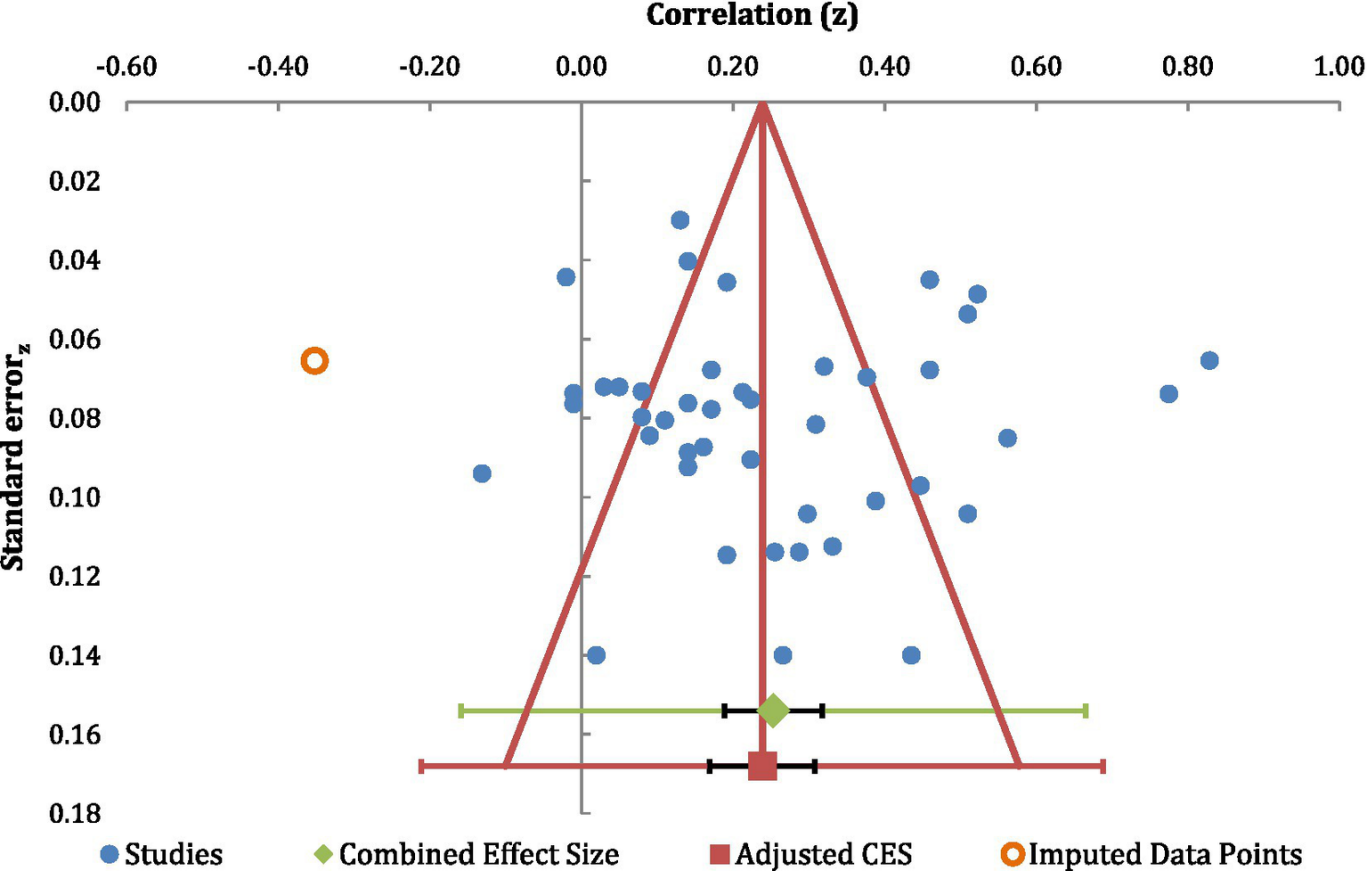
A funnel-plot examining publication bias in the correlational analysis.

**Figure 5:**
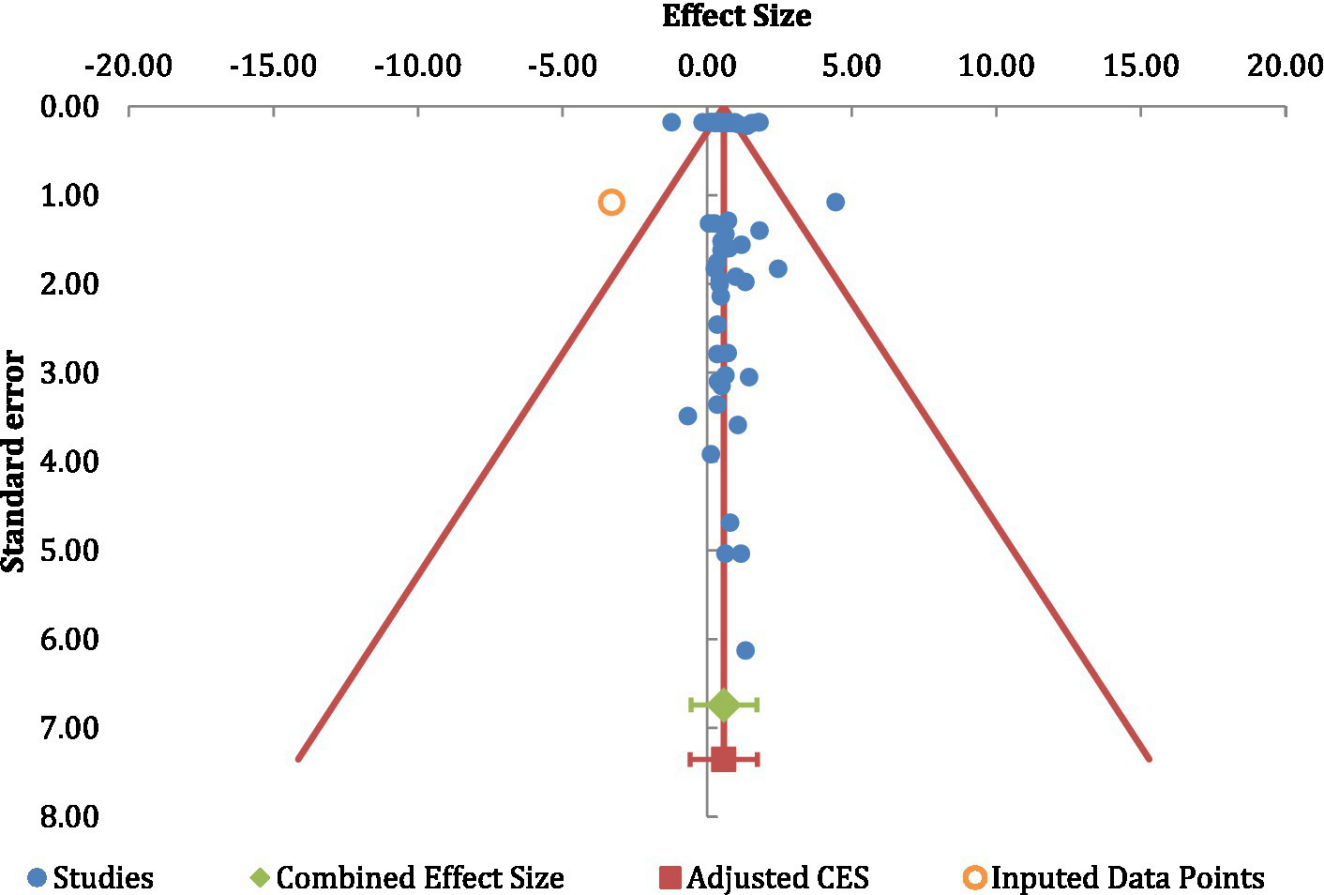
A funnel-plot examining publication bias in the pairwise analyses.

To address the potential for any inherent publication bias, we constructed two funnel plots derived from our data. Here, we employed the “trim-and-fill” method advocated by Duval and Tweedie (2000) (see also Schmidt and Hunter, 2015). As can be seen in Figure 4 for the correlation data, and Figure 5 for the pairwise analysis, there was little evidence of any such publication bias in either case. In addition, for the correlational analysis, the trimmed mean effect size (0.24) was very similar to the unadjusted value (0.25). Also, the trimmed mean for the pair-wise analysis (0.57) was also very similar to that for the unadjusted value (0.58). If publication biases were present in this area of study, it would be expected that the trimmed values should be manifestly different from the unadjusted values (and see Schmidt and Hunter, 2015).

## Meta-analysis results

### Trustor factors

Specific predictive factors were identified and examined separately to determine their effect**s** on the three areas of examined trust (i.e., trustworthiness, propensity to trust, and general trust). Trustworthiness was further sub-divided into a downward direction (trustworthiness of a subordinate) and an upward direction (trustworthiness of a supervisor). Trustor factors included all variables that applied to the person who was doing the trusting, i.e., the person who was placing themselves in a position of potential vulnerability. So here the identified ability variables applied to the trustor, not of the person in whom they were placing their trust. Not all factors, identified in the present descriptive structure, were found in the literature pertaining to each analysis. Since our meta-analytic review drew from the whole body of work concerning trust, and not all articles identified provided sufficient information to determine effect sizes, some independent and/or predictor variables were found in the literature for one directional relationship but not for another. For instance, a variable might have been found to correlate to trustor factors, but not found in any articles referencing those factors for trustees. Identification of these lacunae help generate recommendations for prospective experimental explorations.

### Trustee factors

The analyses of trustee factors examined aspects of the person in whom trust is being placed. For example, the trustee independent and/or predictor variable of “gender” refers exclusively to whether the trustee identified as male or female. The level of trust or perceived trustworthiness however is still reported by the trustor.

### Contextual factors

Contextual or environmental factors, serve to affect both trustor and trustee. These factors pertain to various aspects of the setting of the trustor-trustee relationship, such as a shared mental model or properties such as team cohesion. Contextual factors are also aspects of the physical environment, or characteristics of the task at hand that affect both trustor and trustee. These can include the presence of objective risk or immediate presence of potential harm such as occur in military operations.

### Overall outcome effects

#### Correlational analysis

We identified 2,125 correlational effect sizes (and see Supplemental material for a complete list of citations and their respective effect sizes). Of the relationships evaluated, the vast majority (89.9%) examined the trustworthiness of another individual as the outcome variable. The individual in question, or the trustee, could be a lateral team member, a supervisor, or a subordinate. A smaller percentage of studies (9.1%) examined an individual’s propensity to trust as a dispositional trait. Ninety-nine percent (99%) of the correlational studies employed at least one of these variables. The remaining ~1% examined general trust and could not be classified as either the propensity to trust or the perception of trustworthiness. Both of the latter were variables of interest and explored further in the present meta-analysis, although perceptions of another’s trustworthiness proved by far the more frequent. The variations across predictor variables was smaller. Thus, 40.5% of correlations looked at trustor factors, 21.7% examined trustee factors, and 37.8% addressed contextual factors that affected both the trustor and trustee together. A more detailed breakdown of the predictor variables is presented in Table 2 below.

**Table 2:** Predictor variables and number of effect sizes derived from each.

Factor in proposed human-human trust model	Number of effect sizes	Effect size %
Trustee trustworthiness	387	9.9
Team cohesion	273	7
Trustor personality traits	244	6.3
Trustor commitment	241	6.2
Trustor-other factors	227	5.8
Shared communication	208	5.3
Shared performance	190	4.9
Trustee personality traits	178	4.6
Other trust measure	155	4
Trustor performance	132	3.4
Shared mental models	120	3.1
Team composition	118	3
Trustor prior experience	110	2.8
Shared task characteristics	104	2.7
Shared factors-other	102	2.6
Team conflict	95	2.4
Shared tenure	88	2.3
Trustor age	86	2.2
Trustee-other factors	80	2.1
Trustor uncertainty	79	2
Trustor gender	75	1.9
Trustor self-efficacy	67	1.7
Shared interdependence	58	1.5
Trustor engagement	39	1
Trustor education	37	1
Trustee performance	37	1
Trustor propensity to trust	34	0.9
Trustee reliability	36	0.9
Trustor culture	32	0.8
Trustee expertise	32	0.8
Shared in group membership	32	0.8
Trustee transparency	26	0.7
Trustee physical appearance	25	0.6
Shared interaction frequency	22	0.6
Trustor race	16	0.4
Trustee reputation	17	0.4
Trustee gender	12	0.3
Trustee age	11	0.3
Trustee trustworthiness, other trust measure	6	0.2
Trustee trustworthiness, trustor other	6	0.2
Shared efficiency	9	0.2
Shared risk	9	0.2
Shared proximity	7	0.2
Team composition, interdependence, and in group membership	8	0.2
Trustee performance, personality	4	0.1
Trustee education	4	0.1
Trustee expertise, reliability	2	0.1
Trustee reliability, reputation	4	0.1
Trustee personality traits, reputation	2	0.1
Shared communication, interaction frequency	2	0.1
Trustee race	1	0
Trustee transparency, reputation, and other	1	0
Team conflict, interdependence, and efficiency	1	0

Individual analyses were conducted between trustworthiness, propensity to trust, general trust, directional trust, and the triad of proposed categories (i.e., trustee, trustor, and contextual factors). Perceptions of trustworthiness represented the largest proportion of the outcome variables and was thus examined first, with trustor factors, trustee factors, and contextual factors serving as the predictor variables. For trustor factors, the overall relationship with trustworthiness proved to be significant (*r =* 0.27). Additionally, trustee factors (*r =* 0.34) and contextual factors (*r =* 0.28) also exhibited similar significant relationships with trustor’s perceptions of trustee’s level of trustworthiness. Of these three factors, proposed to predict trust development, none of the associated 95% confidence intervals contained the zero value. This supports a conclusion that trustworthiness is to some degree associated with all three of these overarching categories. That is, the interpersonal trust between individuals can be derived, at least to some consistent extent, from each of the trustor, the trustee as well as contextual situation in which they interact. More information, such as confidence intervals and the number of the samples surveyed, are specified in Table 3. For each of the average weighted correlations shown in Table 3, the proportion of observed variance that is due to sampling error was estimated to be below the Hunter and Schmidt (2004) 0.75 threshold for homogeneity. That is, in each case, there remains a degree of systematic variance in the correlations that can be explored using further moderator analyses.

**Table 3:** Overall trust predictors.

Factor category	Trust category	*K*	*r*	$s_{g}^{2}$	$s_{g}^{2}$	se2sg2	95% confidence interval	
							Lower limit	Upper limit
Trustee factors	Trustworthiness	76	0.34^*^	0.004	0.07	0.06	0.33	0.36
Contextual factors	Trustworthiness	141	0.28^*^	0.06	0.01	0.09	0.27	0.29
Trustor factors	Trustworthiness	136	0.27^*^	0.003	0.05	0.07	0.26	0.28
Trustee factors	Propensity to trust	17	0.16^*^	0.01	0.02	0.46	0.12	0.21
Trustor factors	General trust	1	0.16^*^	—	—	—	—	—
Contextual factors	Propensity to trust	23	0.13^*^	0.007	0.013	0.55	0.09	0.21
Trustor factors	Propensity to trust	43	0.10^*^	0.003	0.01	0.36	0.08	0.12
Contextual factors	General trust	1	0.15	—	—	—	—	—
								
Trustor predictors	Trust category	*K*	*r*	$s_{g}^{2}$	$s_{g}^{2}$	se2sg2	95% confidence interval	
							Lower limit	Upper limit
**Trustor abilities**								
Commitment	Trustworthiness	33	0.44^*^	0.005	0.05	0.10	0.42	0.47
	Propensity to trust	14	0.19^*^	0.01	0.02	0.38	0.15	0.23
Engagement	Trustworthiness	9	0.40^*^	0.001	0.06	0.02	0.38	0.42
	Propensity to trust	2	0.36^*^	0.01	0.00	--	0.23	0.49
Performance	Trustworthiness	22	0.28^*^	0.01	0.07	0.11	0.24	0.32
	Propensity to trust	9	0.18^*^	0.01	0.02	0.41	0.12	0.24
Uncertainty	Trustworthiness	16	0.05^*^	0.01	0.11	0.08	0.01	0.10
	Propensity to trust	2	−0.06	0.01	0.003	3.35	−0.21	0.10
Prior experience	Trustworthiness	14	0.00	0.003	0.007	0.46	−0.03	0.03
	Propensity to trust	8	0.08^*^	0.01	0.003	3.01	0.02	0.15
**Characteristics**								
Other trust measures	Trustworthiness	35	0.47^*^	0.01	0.07	0.09	0.45	0.50
	Propensity to trust	3	0.09	0.01	0.03	0.38	−0.02	0.21
Self-efficacy	Trustworthiness	18	0.36^*^	0.01	0.03	0.31	0.32	0.40
	Propensity to trust	2	0.32^*^	0.01	0.02	0.45	0.21	0.44
Personality traits	Trustworthiness	38	0.25^*^	0.003	0.04	0.08	0.24	0.27
	Propensity to trust	19	0.08^*^	0.002	0.01	0.16	0.06	0.10
Propensity to trust	Trustworthiness	10	0.22^*^	0.01	0.01	1.01	0.16	0.29
	Propensity to trust	7	0.17^*^	0.02	0.01	1.55	0.06	0.27
Culture	Trustworthiness	6	0.22^*^	0.01	0.16	0.06	0.14	0.30
	Propensity to trust	8	0.10^*^	0.004	0.08	0.05	0.06	0.15
Gender	Trustworthiness	19	0.10^*^	0.002	0.02	0.12	0.08	0.12
	Propensity to trust	8	0.04^*^	0.002	0.003	0.57	0.02	0.07
	General trust	1	−0.07	—	—	—	—	—
Age	Trustworthiness	26	0.07^*^	0.003	0.007	0.35	0.05	0.09
	Propensity to trust	11	0.10^*^	0.002	0.00	2.02	0.08	0.13
	General trust	1	0.23^*^	—	—	—	—	—
Race	Trustworthiness	3	−0.02	0.01	0.005	1.19	−0.10	0.07
	Propensity to trust	3	−0.11^*^	0.003	0.001	3.18	−0.18	−05
Education	Trustworthiness	7	−0.06	0.006	0.014	0.46	−0.12	0.00
	General trust	1	0.06	—	—	—	—	—
	Propensity to trust	6	0.15^*^	0.01	0.02	0.32	0.09	0.21
Other factors	Trustworthiness	32	0.17^*^	0.004	0.04	0.10	0.15	0.19
	Propensity to trust	7	0.17^*^	0.004	0.005	0.75	0.13	0.22
	General trust	1	0.70	—	—	—	—	—
Global	Trustworthiness	136	0.27^*^	0.003	0.05	0.07	0.26	0.28
	Propensity to trust	43	0.10^*^	0.003	0.01	0.36	0.08	0.12
	General trust	1	0.16	—	—	—	—	—
Trustee predictors	Trust category	*K*	*r*	$s_{g}^{2}$	$s_{g}^{2}$	se2sg2	95% confidence interval	
							Lower limit	Upper limit
**Trustee abilities**								
Expertise	Trustworthiness	14	0.41^*^	0.01	0.07	0.10	0.37	0.46
	Propensity to trust	1	0.17	—	—	—	—	—
Reliability	Trustworthiness	11	0.32^*^	0.004	0.10	0.05	0.28	0.36
	Propensity to trust	2	0.13^*^	0.01	0.00	8.34	0.03	0.23
Performance	Trustworthiness	7	0.28^*^	0.01	0.04	0.22	0.21	0.35
	Propensity to trust	1	0.30	—	—	—	—	—
**Characteristics**								
Transparency	Trustworthiness	2	0.48^*^	0.00	0.00	14.52	0.42	0.54
	Propensity to trust	1	0.12	—	—	—	—	—
Trustworthiness	Trustworthiness	49	0.47^*^	0.003	0.03	0.12	0.46	0.49
	Propensity to trust	12	0.20^*^	0.01	0.03	0.46	0.14	0.27
Reputation	Trustworthiness	4	0.27^*^	0.004	0.014	0.32	0.21	0.34
Race	Trustworthiness	1	0.22	—	—	—	—	—
Physical appearance	Trustworthiness	8	0.11^*^	0.01	0.03	0.20	0.05	0.16
Personality traits	Trustworthiness	9	0.01	0.01	0.30	0.02	−0.05	0.07
	Propensity to trust	4	0.07	0.010	0.014	0.71	−0.03	0.17
Age	Trustworthiness	4	0.01	0.01	0.04	0.23	−0.09	0.10
Gender	Trustworthiness	2	−0.02	0.002	0.004	0.61	−0.10	0.05
Other trustee variables	Trustworthiness	4	−0.14^*^	0.01	0.02	0.30	−0.21	−0.06
Global	Trustworthiness	76	0.34^*^	0.004	0.07	0.06	0.33	0.36
	Propensity to trust	17	0.16^*^	0.01	0.02	0.46	0.12	0.21
Contextual predictors	Trust category	*K*	*r*	$s_{g}^{2}$	$s_{g}^{2}$	se2sg2	95% confidence interval	
							Lower limit	Upper limit
**Collaborative**								
In-group membership	Trustworthiness	13	0.57^*^	0.003	0.23	0.02	0.54	0.61
	Propensity to trust	2	0.07	0.01	0.0006	18.39	−0.08	0.21
Shared mental models	Trustworthiness	33	0.42^*^	0.004	0.05	0.08	0.40	0.44
	Propensity to trust	4	0.23^*^	0.01	0.04	0.28	0.12	0.34
Team cohesion	Trustworthiness	44	0.37^*^	0.01	0.04	0.22	0.34	0.40
	Propensity to trust	11	−0.44^*^	0.01	3.39	0.00	−0.50	−0.38
Communication	Trustworthiness	42	0.34^*^	0.01	0.07	0.10	0.32	0.37
	Propensity to trust	3	0.03	0.009	0.012	0.77	−0.08	0.14
	General trust	1	0.19	—	—	—	—	—
Interdependence	Trustworthiness	15	0.28^*^	0.01	0.05	0.14	0.24	0.32
	Propensity to trust	4	0.07	0.01	0.02	0.41	−0.02	0.15
Performance	Trustworthiness	56	0.27^*^	0.01	0.04	0.27	0.24	0.30
	Propensity to trust	4	0.10	0.02	0.01	1.67	−0.03	0.23
	General trust	1	0.32	—	—	—	—	—
Shared efficiency	Trustworthiness	1	0.24	—	—	—	—	—
	Propensity to trust	3	0.22^*^	0.003	0.002	1.21	0.16	0.28
Interaction frequency	Trustworthiness	10	0.16^*^	0.012	0.008	0.65	0.11	0.22
	Propensity to trust	2	0.17	0.01	0.03	0.54	0.00	0.33
Shared tenure	Trustworthiness	22	0.10^*^	0.011	0.014	0.75	0.06	0.14
	Propensity to trust	4	0.01	0.01	0.03	0.44	−0.10	0.12
	General trust	1	0.05	—	—	—	—	—
Proximity	Trustworthiness	3	0.10	0.01	0.02	0.52	−0.02	0.22
Team composition	Trustworthiness	36	0.05^*^	0.01	0.02	0.76	0.02	0.09
	Propensity to trust	4	0.00	0.009	0.013	0.74	−0.10	0.10
Risk	Trustworthiness	3	−0.04	0.003	0.03	0.13	−0.11	0.02
Team conflict	Trustworthiness	31	−0.25^*^	0.01	0.08	0.12	−0.28	−0.21
	Propensity to trust	2	−0.10	0.008	0.001	6.67	−0.23	0.02
**Tasking factors**								
Task characteristics	Trustworthiness	34	0.16^*^	0.01	0.04	0.18	0.13	0.19
	Propensity to trust	3	0.01	0.02	0.01	1.51	−0.13	0.16
Other factors	Trustworthiness	27	0.22^*^	0.01	0.13	0.08	0.27	0.29
	Propensity to trust	2	0.00	0.02	0.03	0.54	−0.19	0.19
Global	Trustworthiness	141	0.28^*^	0.06	0.01	0.09	0.54	0.61
	Propensity to trust	23	0.13^*^	0.007	0.013	0.55	0.09	0.21
	Propensity to trust	23	0.13^*^	0.007	0.013	0.55	0.09	0.21

* Significant beyond the *p* < 0.05 level.

$s_{g}^{2}$, sampling error variance; $s_{g}^{2}$, observed variance.

For the outcome variable of trustor propensity to trust, the same three overall categories were examined. Trustor factors showed a weak correlation with propensity to trust (*r =* 0.1). The confidence interval for these trustor factors, however, still did not contain a value of zero. This result indicates that the identified relationship is consistent, albeit a small one. Trustee factors related to the propensity to trust also showed a significant correlation (*r =* 0.16), as again did contextual factors (*r =* 0.13). Though an individual’s propensity to trust does not show as strong a relationship to any of the categories as did perceptions of trustworthiness, the influence of trustor, trustee, and contextual factors remains consistent and quantitatively demonstrable. All of these findings are enumerated in Table 3. For most of the correlations shown in Table 3 showing the trustor predictor variable, the proportion of total variance due to sampling error is below 0.75, suggesting heterogeneity of the effect sizes. However, in some illustrated cases, the proportion is greater than 1. If there are several studies contributing to this effect size estimate, this can be interpreted to mean that the effect sizes are homogeneous (e.g., see the correlation between propensity to trust and trustworthiness).

From the surveyed literature, we were able to define general trust in instances where trust could be described neither as an individual’s propensity to trust nor as their perception of another’s trustworthiness. The relationship between this categorization of general trust and trustor factors did show a significant correlation (*r =* 0.16). Here, again the associated confidence interval here did not include zero. The relationship between general trust and contextual factors (*r =* 0.15) did not, however, prove to be significant since it did contain zero in the related confidence interval. There were no code-able studies identified that included a correlation between trustee factors and general trust. That is not to say that such trustee factors are not associated with general trust, only that our present search was not able to identify any admissible data so as to determine whether such an effect exists.

The decomposition of variables thus far is informed by, and guided by, both the overall trust literature and the model of human-interpersonal trust that we have previously offered (Hancock et al., 2011b; Schaefer et al., 2016; and see also Hancock et al., 2021a,b). However, the three general predictor categories can themselves be further sub-divided. Contextual factors, for example, are able to be parsed into two sub-categories: (i) task-based characteristics (e.g., task type, task complexity, and level of uncertainty/risk) and (ii) collaborative factors (e.g., in-group membership, communication, interaction conflict, and role interdependence). Trustee dimensions are also able to be further parsed into two sub-categories: (i) trustee characteristics (e.g., demographics, personality, appearance, and reputation), and (ii) trustee performance (e.g., behavior, reliability, predictability). Trustor factors were similarly divided by these self-same characteristics and performance differentiations.

Of the many identified variables, one that proved to be of particular interest was gender. Many studies examined correlations between gender (of both trustor and trustee) and trust. Although gender was coded differently in many studies, we were careful to assure that all of the correlations used in our meta-analysis were consistent. Thus, males were always coded as 0 and females as 1. With this standardization completed, our analysis showed that the relationship between gender and general trust (*r =* −0.07) was not significant, this effect containing zero within its confidence interval. Both the relationships between gender and propensity to trust (*r =* 0.04) and gender and perceptions of trustworthiness (*r =* 0.1) also proved small and non-significant.

### Discussion of the overall pattern of correlations

**Figure 6:**
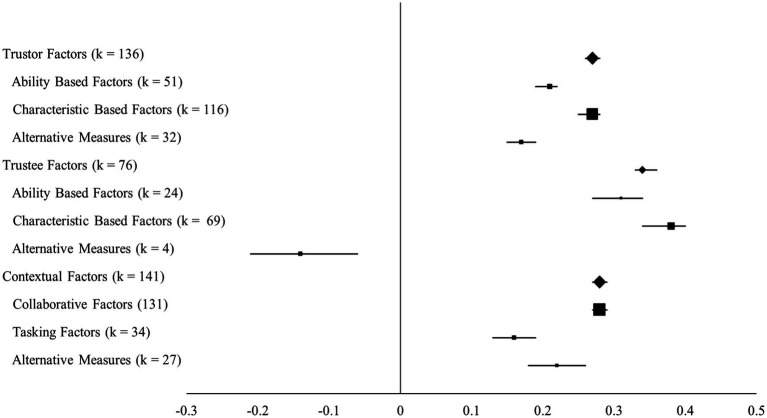
Forest plot of factors found to affect trustworthiness. Line length represents the boundaries of each 95% confidence interval. Diamonds represent global effects, whereas squares represent category effects. The size of each diamond and square represents the size of each respective effect with larger shapes representing larger effects.

Overall correlations indicated that trustor factors, trustee factors, and contextual factors all demonstrated significant and moderately sized relationships with perceived trustworthiness. Trustor, trustee, and contextual factors also exerted significant but quantitatively smaller correlations with the propensity to trust. Trustor and contextual factors had significant but small correlations with general trust. As these proposed factors represent over-arching groupings composed of more specific characteristics, those factors which showed significant correlations with trustworthiness, propensity to trust, and general trust were divided into specific characteristics (see Figure 6). These are now examined individually in more detail.

## Trustor factors

The following analysis illustrates the relationship between trustor variables, and several dimensions of trust, including trustworthiness, or the trustor’s rating of another individual’s trustworthiness; the trustor’s propensity to trust; and general trust, which could not be defined in any other way (see Table 3). There were two statistically significant variables of interest that correlated with a trustor’s sense of another individual’s trustworthiness. These were the trustor’s culture (*r =* 0.22) and the trustor’s performance (either self-rated or observed performance during the same task where they rated their trust; *r =* 0.28). Each were related to the extent to which they considered another person trustworthy. Significant correlations of interest also included those between a trustor’s propensity to trust, and their level of commitment (*r =* 0.19), their engagement (*r =* 0.36) as well as their self-efficacy (*r =* 0.32). Although there were a number of other significant correlations, it appears that each of these variables involve an individual’s sense of ability and obligation, and they are all moderately to strongly correlated with that individual’s propensity to trust. Also, propensity to trust was correlated moderately (*r =* 0.22) with their perception of the trustee’s trustworthiness.

## Trustee factors

Analysis for trustee factors quantified the relationship between trustee variables, and the trustor’s rating of said trustee’s trustworthiness. The predictor variables each relate to the trustee, but the trustor remains the one who rates their own level of trust (Table 3).

The relationship between trustee traits, and perceived trustworthiness of the trustee proved to be a strong one. It is of little surprise that trustee expertise (*r =* 0.41), performance (*r =* 0.28), reliability (*r =* 0.32), reputation (*r =* 0.27), and transparency (*r =* 0.48) are each significantly and positively correlated with their perceived trustworthiness. A trustee’s race also played a role (*r =* 0.22) in determining how trustworthy they appeared. An individuals’ trustworthiness was, as expected, also highly correlated with their perceived trustworthiness (*r =* 0.47). Though it may initially appear to be surprising to correlate two seemingly identical traits, trustworthiness is operationalized as their integrity as shown or measured in another fashion while the outcome variable of perceived trustworthiness is measured *via* the trustor’s self-reported trust in the trustee. Although these relationships were strong, the proportion of observed variance accounted for by sampling error indicated a substantial degree of heterogeneity of effect sizes. It is thus likely that even here there remain other factors that still moderate these observed effects.

## Contextual factors

Contextual factors are those that are shared by both the trustor and the trustee. These include both task characteristics and common environmental considerations. The quantitative results regarding these contextual factors are reported here in Table 3.

Team communication and perceived trustworthiness of the trustee were significantly correlated (*r =* 0.34) as were trustworthiness and in-group membership (*r =* 0.57), as well the presence of shared mental models (*r =* 0.42). Team cohesion was positively correlated with perceived trustworthiness (*r =* 0.37), and team conflict was again, not unexpectedly, negatively correlated therewith (*r =* −0.25). Many of these contextual antecedents of trust are to be expected since they deal with the unity of a team, and perceived trustworthiness of a teammate is a natural derivative of any team’s unity or division. Finally, the influence of trustee factors was found to have the largest relationships with trustworthiness. Specifically, the characteristics that the trustee possesses were found to be the biggest influencer of trustworthiness in our overall model (and see Figure 6). The proportions of variance indicate that these effects are heterogeneous, with one exception. The correlation between team composition and trustworthiness was associated with a variance proportion of 0.76. Based on Hunter and Schmidt’s criterion, this suggests that the remaining 0.24 of the variance is also likely due to forms of statistical artifact. In other words, the effect sizes contributing to this purported influence can be considered homogeneous. A similar argument can be made for the correlation between shared tenure and trustworthiness.

## Directional trust

A subset of studies assessed here involved the directionality of trust. For example, upward trust is typically associated with one’s interaction with a supervisor while downward trust is often expressed as related to that with a subordinate. This issue of directionality is primarily one of power differential. Such relationships involve a trustor with some evident and explicit power over a subordinate and a trustor most often in a subservient position. One can also argue that, to at least some degree, power differentials are involved in all trusting activities, being inherent in all risk exposures. The reported relationships, however, were explicitly observed between the traits of a trustor and their perception of a subordinate’s, or supervisor’s trustworthiness. The supervisor here can be characterized as a manager, commanding officer, chief surgeon, or in some manner an individual higher in the chain of command compared to the other person in the trust relationship (Alston and Tippett, 2009; Zand, 1997). Such power differentials also inhere in families and other associated social units. These studies were also included in overall correlations.

### Downwards trust

The following factors were examined to determine their influence on trust in a subordinate and these results are shown in Table 4.

**Table 4:** Directional trust.

Trust antecedent	Predictor type	*K*	*r*	$s_{e}^{2}$	$s_{g}^{2}$	se2sg2	95% confidence interval	
							Lower limit	Upper limit
Trust in Subordinate								
Engagement	Trustor	2	0.46^*^	0.01	0.02	0.43	0.32	0.60
Performance	Trustor	4	0.36^*^	0.01	0.08	0.16	0.25	0.47
	Trustee	12	0.38^*^	0.01	0.03	0.25	0.33	0.43
	Contextual	7	0.21^*^	0.009	0.015	0.61	0.14	0.28
Trustworthiness	Trustee	8	0.35^*^	0.01	0.02	0.62	0.28	0.42
Reliability	Trustee	2	0.58^*^	0.003	0.01	0.32	0.50	0.67
Commitment	Trustor	6	0.30^*^	0.008	0.01	0.81	0.23	0.38
Uncertainty	Trustor	2	0.23^*^	0.01	0.004	3.01	0.07	0.39
Other	Trustor	6	0.22^*^	0.01	0.10	0.08	0.15	0.29
	Trustee	12	0.24^*^	0.01	0.05	0.21	0.18	0.29
	Contextual	6	0.12^*^	0.01	0.05	0.24	0.03	0.21
Other trust measure	Trustor	3	0.20^*^	0.01	0.09	0.10	0.09	0.31
Education	Trustor	1	0.16	—	—	—	—	—
	Trustee	1	0.03	—	—	—	—	—
Expertise	Trustee	2	−0.02	0.02	0.01	1.11	−0.20	0.15
Age	Trustor	1	0.13	—	—	—	—	—
	Trustee	2	0.12	0.01	0.01	0.93	−0.02	0.26
Propensity to trust	Trustor	1	0.10	—	—	—	—	—
Gender	Trustor	1	0.03	—	—	—	—	—
	Trustee	3	−0.06	0.01	0.003	4.18	−0.18	0.06
Race	Trustor	1	−0.05	—	—	—	—	—
Prior experience	Trustor	3	−0.06	0.014	0.006	2.16	−0.19	0.08
Personality	Trustor	1	−0.11	—	—	—	—	—
	Trustee	4	0.14	0.01	0.01	0.67	0.05	0.24
Reputation	Trustee	2	0.18	0.01	0.04	0.23	0.05	0.31
Culture	Trustor	2	−0.24^*^	0.01	0.00	60.88	−0.40	−0.08
Shared mental models	Contextual	2	0.39^*^	0.01	0.11	0.08	0.27	0.52
Communication	Contextual	5	0.35^*^	0.01	0.04	0.23	0.26	0.43
Team cohesion	Contextual	1	0.31^*^	—	—	—	—	—
Team composition	Contextual	2	0.26^*^	0.02	0.05	0.35	0.07	0.45
Task characteristics	Contextual	2	0.21^*^	0.008	0.004	2.11	0.08	0.34
Efficiency	Contextual	1	0.08	—	—	—	—	—
Shared tenure	Contextual	10	0.05	0.009	0.013	0.72	−0.01	0.11
Interdependence	Contextual	1	−0.04	—	—	—	—	—
Risk	Contextual	1	−0.13	—	—	—	—	—
Team conflict	Contextual	1	−0.18	—	—	—	—	—
Trust in a Supervisor								
Engagement	Trustor	17	0.52^*^	0.003	0.004	0.08	0.50	0.55
Other trust measures	Trustor	17	0.52^*^	0.003	0.05	0.07	0.49	0.55
Self-efficacy	Trustor	22	0.28^*^	0.01	0.03	0.20	0.25	0.31
Commitment	Trustor	48	0.27^*^	0.01	0.05	0.13	0.25	0.30
Personality traits	Trustor	37	0.25^*^	0.01	0.06	0.11	0.22	0.28
	Trustee	36	0.52^*^	0.004	0.08	0.05	0.50	0.54
Performance	Trustor	59	0.20^*^	0.01	0.02	0.45	0.18	0.23
	Trustee	2	0.45^*^	0.01	0.02	0.50	0.32	0.58
Propensity to trust	Trustor	7	0.18^*^	0.02	0.03	0.54	0.09	0.27
Other	Trustor	40	0.15^*^	0.01	0.07	0.10	0.12	0.18
	Trustee	13	0.21	0.01	0.06	0.18	0.15	0.26
Education	Trustor	16	0.07^*^	0.01	0.01	0.98	0.02	0.11
Uncertainty	Trustor	18	0.06^*^	0.01	0.05	0.15	0.02	0.10
Culture	Trustor	4	0.09	0.01	0.02	0.56	0.00	0.19
Age	Trustor	30	0.00	0.009	0.01	0.89	−0.03	0.04
Prior experience	Trustor	34	0.00	0.007	0.006	1.12	−0.03	0.03
Gender	Trustor	31	−0.02	0.009	0.007	1.32	−0.05	0.01
	Trustee	2	0.05	0.02	0.003	4.18	−0.12	0.22
Race	Trustor	3	−0.05	0.009	0.013	0.66	−0.16	0.05
Expertise	Trustee	5	0.57^*^	0.004	0.11	0.04	0.52	0.63
Reliability	Trustee	11	0.57^*^	0.005	0.02	0.22	0.52	0.62
Trustworthiness	Trustee	39	0.56^*^	0.003	0.05	0.07	0.54	0.58
Reputation	Trustee	9	0.52^*^	0.01	0.05	0.20	0.46	0.59
Transparency	Trustee	8	0.45^*^	0.005	0.019	0.28	0.40	0.50
Efficiency	Contextual	1	0.48	—	—	—	—	—
Team cohesion	Contextual	21	0.44^*^	0.004	0.03	0.16	0.41	0.47
Communication	Contextual	38	0.37^*^	0.01	0.06	0.09	0.35	0.40
Shared mental models	Contextual	13	0.31^*^	0.01	0.06	0.10	0.27	0.35
Interaction frequency	Contextual	3	0.28^*^	0.004	0.01	0.67	0.20	0.36
Performance	Contextual	14	0.27^*^	0.01	0.03	0.32	0.22	0.32
Other	Contextual	13	0.25^*^	0.01	0.04	0.16	0.21	0.30
Shared risk	Contextual	4	0.25^*^	0.01	0.03	0.39	0.14	0.36
Interdependence	Contextual	6	0.22^*^	0.004	0.07	0.06	0.17	0.27
Team conflict	Contextual	8	−0.15^*^	0.004	0.09	0.06	−0.19	−0.10
Task characteristics	Contextual	10	0.14^*^	0.01	0.06	0.10	0.09	0.19
Team composition	Contextual	3	0.07	0.01	0.02	0.45	−0.05	0.18
Shared tenure	Contextual	10	0.05	0.009	0.01	0.72	−0.01	0.11
In-group membership	Contextual	6	−0.01	0.01	0.14	0.08	−0.09	0.07

* Significant beyond the *p* < 0.05 level.

$s_{e}^{2}$, sampling error variance; $s_{g}^{2}$, observed variance.

When examining power distance in trustor-trustee relationship, our summed results showed significant relationships between the trustor’s engagement (*r =* 0.46), performance (*r =* 0.36), uncertainty (*r =* 0.23), commitment (*r =* 0.30), and culture (*r =* −0.24). These proved influential, as well as other variables, that did not fall under any of the predicted categories, (*r =* 0.22), or other noted trust measures (*r =* 0.20).

**Figure 7:**
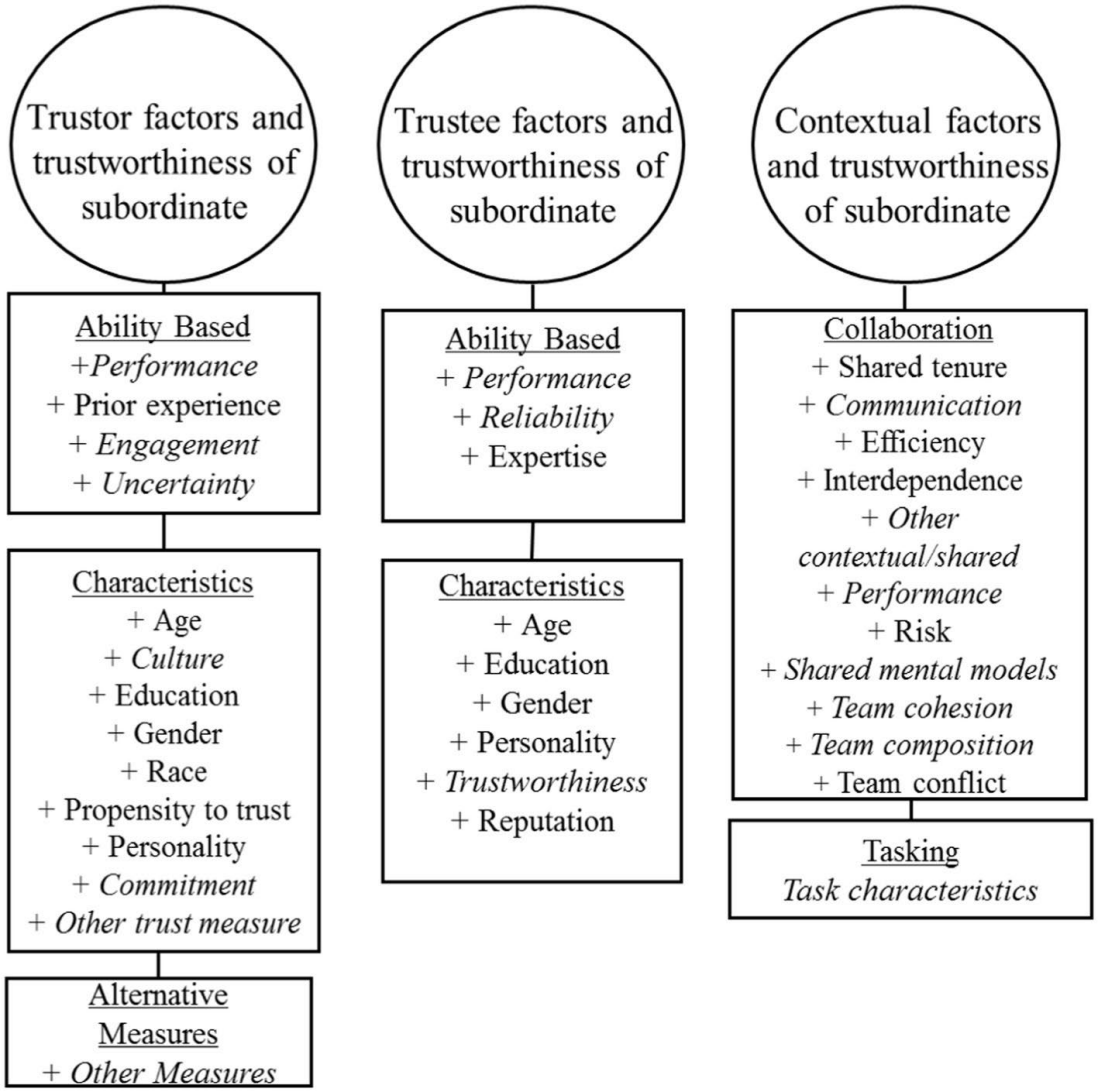
Correlational and experimental findings for directional trust. Trust in a subordinate (downward trust) originated from the supervisor or trustor report of themselves and their report of their subordinate. Trust in a supervisor (upwards trust) originated from the subordinate or trustor report of themselves and their report of their supervisor. Correlational evidence is marked with a +. Significance is flagged with italics.

When the trustee was a subordinate, their performance (*r =* 0.38), reliability (*r =* 0.58), and trustworthiness (*r =* 0.35) were identified as significant predictors of how much their superior trusted them. Finally, the contextual factors of shared communication (*r =* 0.35), shared performance (*r =* 0.21), shared mental model (*r =* 0.39), team cohesion (*r =* 0.31), task characteristics (r *=* 0.21), team composition (*r =* 0.26), and the residual, or “other” category (*r =* 0.12) were all significantly related to trustworthiness of a subordinate. Each of these identified relationships are illustrated in Figure 7. Although most of the variance proportions indicate heterogeneity, there were nevertheless exceptions. The correlations between trust in a subordinate and commitment, and trust in a subordinate and age, each were associated with variance proportions above 0.75, again indicating homogeneity of effect sizes.

### Upwards trust

**Figure 8:**
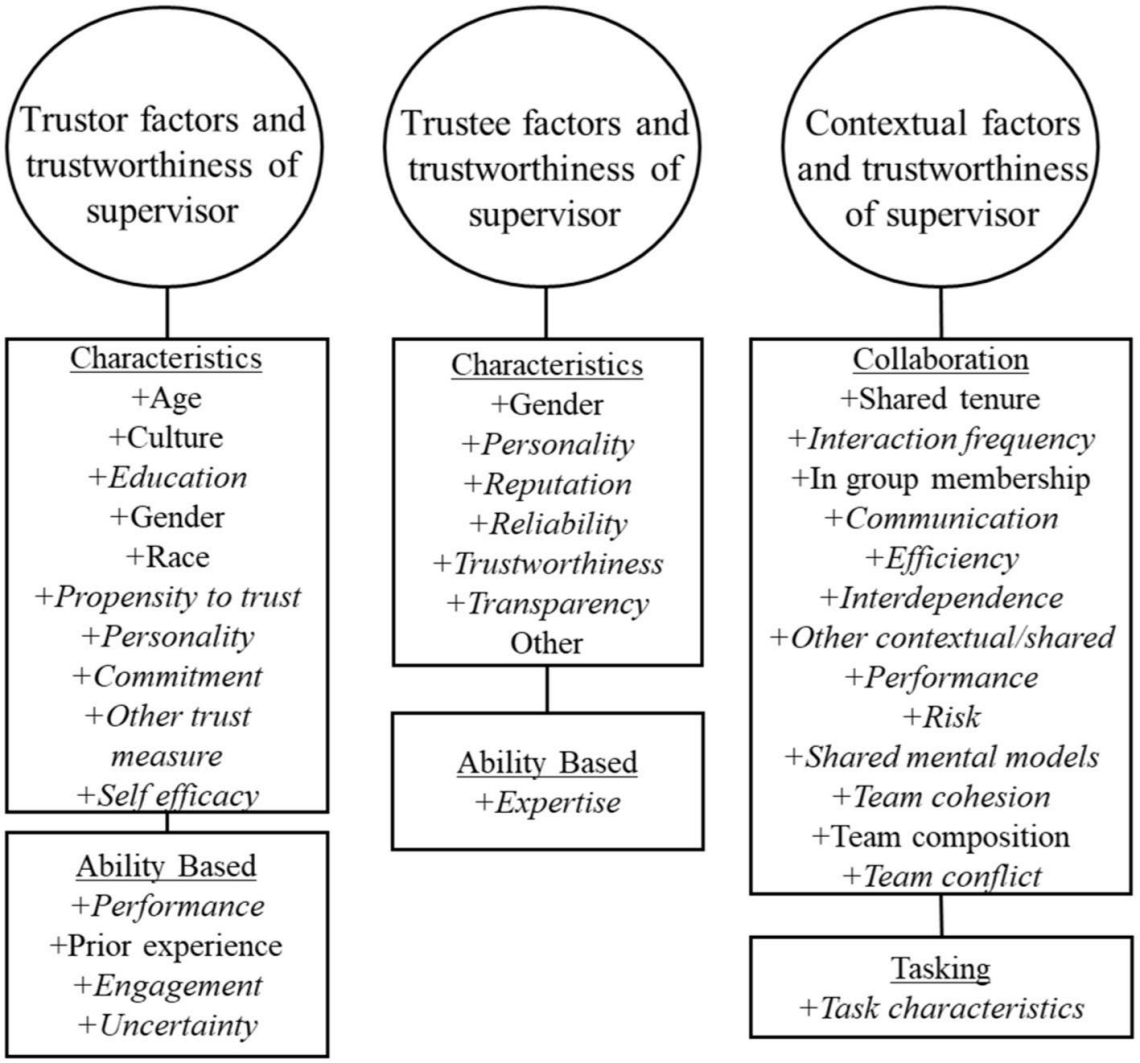
Factors found which influence trustworthiness. Terms with a (+) represent correlational findings, italicized terms represent significant correlational findings. Terms with a (^*^) represent experimental findings, bolded items represent significant experimental findings.

A supervisor could be anyone with power over the trustor or trustee. These relationships were also included in the overall trust assessment above but were examined separately here and reported in Table 4 and Figure 8.

### Discussion of trustworthiness of a supervisor

When examining their trust in a supervisor, the subordinate’s engagement (*r =* 0.52), their commitment (*r =* 0.27), the degree of associated uncertainty (*r =* 0.06), level of education (*r =* 0.07), perceived performance efficiency (*r =* 0.20), personality traits (*r =* 0.25), propensity to trust (*r =* 0.18), and self-efficacy (*r =* 0.28), as well as other trust measures (*r =* 0.52), and the residual category (*r =* 0.15) all proved to be significantly related to their trust in a superior. When the trustee was a superior, we found that their subordinates’ trust increased with the supervising trustee’s expertise (*r =* 0.57), their performance (*r =* 0.45), their personality traits (*r =* 0.52), their reliability (*r =* 0.57), their reputation (*r =* 0.52), the transparency of their interaction (*r =* 0.45), and, finally, their trustworthiness (*r =* 0.56). The examination of contextual factors showed that communication (*r =* 0.37), efficiency (*r =* 0.48), interaction frequency (*r =* 0.28), interdependence (*r =* 0.22), shared mental model (*r =* 0.31), performance (*r =* 0.27), risk (*r =* 0.25), task characteristics (*r =* 0.14), team cohesion (*r =* 0.44), and the residual category (*r =* 0.25) were all significantly and positively related to trustworthiness of a supervisor. Team conflict (*r =* −0.15) was significantly and negatively related to that same characteristic. These collective findings are illustrated graphically in Figure 7. The majority of these effects are heterogeneous, with the two exceptions of the correlation between upwards trust and education of the trustor and upwards trust and age of the trustor. In each of these latter two cases, the variance proportion exceeded 0.75.

## Experimental analyses

### Pairwise comparisons

As might be suspected, given the nature of the various disciplines from which the present information has been drawn, there were far fewer qualifying works that used experimental approaches as compared to those reporting correlational studies. The experimental studies examined the dependent variable of trustworthiness predominantly by using pairwise comparisons. Although the topics that were studied covered rather disparate areas, there were several common categories into which results could be fixed. These categories were (i) attractiveness (high versus low), (ii) closeness (high versus low), (iii) cooperative tendencies (cooperative versus individualistic), (iv) experience (more versus less), (v) trustee gender (female versus male), (vi) trustor gender (female versus male), (vii) group (in-group versus out-group), (viii) presence (in person versus online), (ix) risk/importance (high versus low), and (x) reputation (good versus poor). Membership of the above identified categories were composed of any studies which reasonably fitted into the specified grouping. For example, in the case of reputation (high versus low), any experimental study which examined some aspect of trust in a high reputation group compared to trust in a lower reputation group was included. Such a grouping ranged from trust in a really, high-reputed individual compared to someone of low-reputation, to even hypothetical scenarios in which participants were asked to rate trust in someone after they had been observed behaving dishonestly as compared to after they had been observed behaving altruistically. Gender specification referred to those situations in which participants were rating their trust in, largely, male vs. female, either as a whole or in a specific case. Trustor gender refers to cases where participants were divided along their own gender lines, and each group was asked to rate the trustworthiness of another human, either male or female. For all of these categories, the first identified condition was anticipated, *a priori*, to be the one inducing higher trust. Thus, in the case of reputation, the high reputation was expected to yield greater trust and *vice-versa*. If the second condition in the category (in this case, the low reputation group) scored higher, then the identified effect size was presented as a negative value. The collective results from this analysis are reported in Table 5.

**Table 5:** Pairwise effect sizes.

Category	*K*	*d*	$s_{e}^{2}$	$s_{g}^{2}$	se2sg2	95% confidence interval	
						Lower	Upper
Trustee gender	6	0.71	9.81	1.50	6.55	−1.79	3.22
Cooperative tendencies	2	0.57	0.73	61.19	0.01	−0.61	1.75
Reputation	17	0.37^*^	0.09	0.66	0.14	0.22	0.51
Risk	4	0.30	0.43	0.02	26.41	−0.34	0.95
Closeness	5	0.22^*^	0.032	0.026	1.23	0.07	0.38
In-group membership	14	0.21	0.18	0.06	2.98	−0.01	0.44
Experience	6	0.08	0.35	0.68	0.51	−0.39	0.55
Presence	3	0.08	0.16	0.11	1.40	−0.37	0.52
Trustor gender	2	−0.02	0.02	0.003	5.47	−0.20	0.17
Attractiveness	1	−0.43	—	—	—	—	—

* Significant beyond the *p* < 0.05 level.

$s_{e}^{2}$ , sampling error variance; $s_{g}^{2}$ , observed variance.

The only two categories here that yielded significant results were reputation and closeness. Neither of these two categories included zero in their associated 95% confidence intervals. Both of these latter effect sizes were positive, being a small effect for closeness and a medium effect for reputation. This confirmed that higher reputation and a greater degree of closeness both served to improve perceptions of trustworthiness. Note, however, that the proportion of variance for reputation indicated a substantial degree of heterogeneity among the effect sizes. Results for other factors also prove informative here. Some categories, such as trustor gender and cooperative tendencies, provide only a small sample size and the confidence interval range was correspondingly broad. Such an outcome encourages us to recommend specific and further experimental investigations into these influences in order to clarify the picture as to their impact.

The estimate of central tendency for these categories remains presently uncertain and so only limited inferences can presently be drawn. But, as stated, the paucity of information with respect to any one of these categories points to fruitful areas for future evaluation. Other potential influences, such as trustee gender, do contain a greater number of studies, but again the outcome range of effects remains wide. It is probable that this pattern derives because such studies are drawn from the rather disparate range of disciplines, as noted in our introduction. Results from other categories, such as grouping (in-group versus out-group), proved to be surprising. In this specific case, it is not the number of included studies *per se*, but again the stability of the associated central tendency that renders a, somewhat surprising, null result. It is always tempting to advocate for further experimental procedures in such cases in order to stabilize these estimates, and of course we are in favor of this strategy. However, some null identifications, e.g., no effect for risk, are especially intriguing and even concerning given the consensus definition of trust. This especially being the case since the appraisal of risk is almost always conceived as being so central to trust in the first place (see Mayer et al., 1995).

### Comparison of correlational and pairwise findings

The present results from the correlational data composed our main analysis. This, rather naturally since it represents the vast majority of the works that met our established inclusion criteria (and see, e.g., Block et al., 2000; Hancock et al., 2007). The basic pattern of findings was, in the main, quite similar between correlational and pairwise comparisons. This, aside from the fact that the pairwise data were evidently much sparser. Even with the general similarity, it is still beneficial to compare and contrast these respective sources of information. The pairwise data, at present, show fewer cases in which significant differences are observed. Indeed, the only significant predictors of trust proved to be reputation and closeness. In both these cases, the effect size was positive, with higher trust being exhibited when two interaction partners were closer (*d* = 0.22), and when the trustee possessed a good reputation (*d* = 0.37). This pattern is similar to the comparative findings from the correlational analyses, where trustee reputation also exerted a significant and positive effect on trust (*r =* 0.27), as did, (for example, a companion of closeness, team cohesion *=* 0.37). The other factors evaluated in the pairwise analysis did not present significance differences. However, in many cases, these trended in the same direction as those for the correlational findings. For instance, although pairwise comparisons of trustee gender had a rather large effect size (*d* = 0.71), the wide confidence intervals show that there was great variance between the findings derived from different studies. In the future, it will also therefore be of import to study other dimensions of outcome distributions beyond central tendency and standardized variability alone (Newell and Hancock, 1984). The correlational result for gender (*r =* −0.02) was smaller and likewise not significant. Attractiveness (*d* = −0.04) did not prove significant in the pairwise analysis, nor was physical appearance (*r =* 0.11) significant in the correlational analysis. Proximity was likewise non-significant in both analyses (i.e., *d* = 0.08, *r =* 0.10, respectively). However, despite these consistent patterns, there remained some evident disparities. Trustor gender was not significant in the pairwise analysis (*d =* −0.02) but was significant in the correlational data (*r =* 0.10), neither effect, however, was particularly strong. Unfortunately, here only two studies qualified to be included in the pairwise analysis of trustor gender. One difference that cannot be so easily explained is the variable of in-group membership. This was a positive, but nonsignificant predictor of trust in the pairwise analysis (*d =* 0.21) but a strongly significant predictor of trust in the correlational data (*r =* 0.57). As this relationship was certainly well-represented in both analytical categories, the difference is surprising. Yet, these two values are not necessarily contradictory, and they are in the same direction. However, one proved significant and the other did not. We consider these various effects in more detail in the overall discussion section below.

## Overall discussion

### Proposed and identified relationships

While the above, specific considerations feature the detailed outcomes across all factors and studies investigated, we now proceed to consider the implications of the major trends in these collective findings. The results of trustworthiness as a dependent variable demonstrated the influence of a variety of trustor and trustee ability-based factors, characteristic-based factors, and other measures which have proven to be significantly related to such trustworthiness. Further, several collaborative factors and one task characteristic were also shown to be related to trustworthiness. Although these findings are not, in and of themselves, especially unexpected, what did come as a surprise was that any trustor’s propensity to trust does not then predict actual trust itself. Although the propensity to trust exhibits a small relationship to the trustworthiness of another, it does not serve to predict that relationship in as strong a fashion as was initially supposed. The lack of strength in this relationship might be explained by the process through which our experiences with others impact, and then override, our own individual initial inclinations to trust. This could imply that the transient dynamics of trust predominate in our immediate affective reactions. This is critical since, in many professional, operational, and domestic situations the phenomenon of “*instant-trust*” (the momentary assessment of trust level upon immediate exposure) may well set a dominantly influential baseline for all subsequent interaction. Thus, there is a dynamic time-course to trust which has, as yet received, insufficient attention. For example, the experimental factors that were found to exert the most significant impact on trustworthiness were trustee reputation and shared closeness between trustor and trustee. These two factors can be conceived of as representing a summary or history of what the individual sees as prior and related experiences. Thus, while reputation provides a generalized conception of others’ experience with that particular trustor, closeness is representative of the trustee’s direct and unmediated personal experience of same. Therefore, somewhat naturally, it is a person’s own experiences, combined with those reported by others, whom we also trust, that sets the scene for our own trust response. These factors sculpt the landscape within which immediate experience is then situated. Consequently, it is these direct experiences, combined with indirect personal assurances upon which we rely to calculate our risk in beginning to deal with any other person or impinging entity.

The propensity to trust, expressed as a dependent variable, exhibited relatively few other moderate or strong relationships. This is likely the case because any one individual’s propensity to trust is more of a personality trait than it is dependent on external forces. The trustor’s own engagement and self-efficacy did serve to predict their propensity to trust. So, it is likely that someone who is higher in the basic propensity to trust is also more likely to be engaged in what they are doing and thus experience a greater degree of self-efficacy. Although there were no factors of the trustee that predicted propensity to trust, the contextual factor of team cohesion did record a significant but negative relationship here. This certainly represents a curious and even counter-intuitive outcome and is one that certainly warrants further investigation. Most especially to establish whether this is a valid effect, or some concern associated with the current and disparate assessment methodologies involved in the qualifying studies. Unlike teams, in which trust is often expressed between individuals at a common power level, differentials in such power levels have been demonstrated to exert consistent effects.

Trust between supervisors and subordinates represents one type of relationship in which the risk factor is contingent upon the evident degree of power differential involved. Several predictive factors were established from the present results, derived specifically from the analyses associated with these constructs. For both directions of the power differential (subordinate vs. supervisor), trustor factors of commitment and engagement each proved to exert moderately strong influences. Further, the trustee factors of performance, reliability, and one’s own trustworthiness were also all strongly related to appraisals of the other’s trustworthiness. Contextual factors that were shared between supervisors and their subordinates, independently serve to influence trust. These were identified as team communication, performance, and shared mental models. Some factors did differ when the trustworthiness of a subordinate was in question. For example, the trustor (supervisor) factors of uncertainty, culture, and performance were all moderate predictors of their own trust in that subordinate. Supervisors also reported that task characteristics, team cohesion, and team composition each moderately affected their trust in their employees. However, when the trustors were subordinates, their manager’s personality and self-efficacy, and their own expertise, personality, reputation, and transparency each served to predict their trust in that supervisor. The contextual factors that predicted the trust of a supervisor included team efficiency, interaction frequency, and interdependence. The fact that different predictors affected downward trust, as compared to upward trust, may be explained at least partially, by the fact that the qualities which make a good and reliable employee are different from those that make a good and reliable supervisor. However, it is also important to observe that every study that was analyzed here, each used what were essentially unique methodological approaches. Thus, not every variable that was examined in studies of upward trust had a corresponding variable in relation to downward trust. Consequently, we presently advocate caution in the interpretation and generalization of the present findings since there may well be variables that are important predictors of downward trust which, to date, have only been studied in relation to upwards trust and *vice-versa*.

The present work has thus identified and explained, to the degree that current results permit, the various factors that influence directional trust. However, as with all meta-analyses, ours is necessarily only a contemporary assessment of such trust and cannot provide a complete anticipation of future developments. In respect of this limitation on forms of punctate assessment, it is important to conduct sequences of meta-analyses in order to establish how the state of understanding evolves across time (Hancock et al., 2021a,b). So, in order to facilitate optimal workplace harmony, further empirical examinations are required to address the current shortfalls in the experimental record. With such reported studies, following meta-analyses can identify how understanding of human trust is updated and elaborated. Subordinates who do not trust their supervisor may find that their work performance suffers. Supervisors who do not trust their subordinates may, in turn, prevent them from expressing their full capacity. Over-trust in either direction also carries risk and that perhaps to the degree that under-trust does. For these reasons, predictors of trust should be fully articulated to permit appropriate calibration. Like trust itself, the understanding of trust is a dynamic process and one which is readily and critically informed by the present pattern of our reported findings.

### In-group and out-group trust

Although our present analysis focuses on the individual level of trust, the impact of our findings on inter-group trust are also of value. Some cultures, or groups, already express an implicit level of trust in members of their own group. This can translate to both social and economic benefits as a result of enhanced in-group cooperation (see Coleman, 1988). Businesses can also function as “networks” in association with other allied companies. These formal and informal systems of collaboration (e.g., *keiretsu*) even allow for in-group relationships between different organizations and institutions (Powell, 1990). In some cases, these mutual understandings can even negate the need for formal legal contracts (Macauley, 1963). And, of course, diverse military branches of any single nation often work in strong cooperation whatever the apparent surface banter as to rivalry. Whether on a broad, country-wide scale, or a more specific company-level, belonging to a group can readily impact trust levels toward all others within that collective, both individuals and groups.

Such a positively skewed initial baseline of trust can, and will, serve as a moderating variable (and see Newell and Hancock, 1984). Clearly, individual participants in each of the studies surveyed here would have expressed different levels of initial trust. This foundation may not have been accounted for by the specifically identified antecedents of trust which were examined and evaluated. Additionally, the positive effects of in-group trust could well have been mirrored by the concomitant negative effects of out-group trust. That is, there are certain pre-conceived notions that can lead an individual to distrust another, based simply on their belonging to two different groups (Hancock, 2015). The dynamics of trust thus possess both fast and slow (acute and chronic) dimensions. Such propensities hold true for example, for religious and ethnic groups (e.g., Tam et al., 2009), as well as the businesses and social groups referenced earlier. Additional factors may also bind any group together, such as vocational affiliation or perceived victimhood (Rotella et al., 2013) among a number of other types of ties. While overall such effects on initial trust might be minimal and so may not affect the results of the controlled empirical studies, it is worth noting that essentially no individual presents a “blank slate” when it comes to trust (Pinker, 2003).

Further, there are variations across personal propensity to trust that may be described, not just as individual differences, but as additional forms of systematic impact. It has been found, for example, that those people who reside in areas with higher heterogeneity and inequality are, in general, less likely to trust (*cf*., Leigh, 2006). This seems to be the case in relation to both ethnic and linguistic heterogeneity. Additionally, inequality of income plays a part in determining trust level (Jordahl, 2009) since those of different socio-economic strata, regardless of what other similarities they share, are sufficiently separate from each other so that they face fundamentally different risks and exhibit fundamentally different needs (Uslaner and Brown, 2005). Although these differences have not been explored with sufficient depth or frequency in order to be used as antecedents of trust in meta-analysis, it is important to consider the ways in which the broader political context of one’s life influences propensity to trust. This is an important concern beyond the manipulations that were made in the controlled studies reported here. So, for example, in money-based trust games, while the payout and risk may be technically equal between partners one player may have, relatively speaking, much more to lose. And if a partner makes the wrong decision, costing the other player a significant tranche of money, their failure may represent only a minor annoyance to a rich player but a devastating loss to a poor one. This consideration of inequality is also necessary in the sub-analyses of trust in supervisors. Often, inequality in business is also perpetuated at the higher levels (Mueller et al., 1989). Thus, authority figures, in many cases, represent not just an out-group member in terms of power over subordinates, but also in terms of dimensions such as race and gender as well. Additionally, as previously noted, each individual has a different level of risk reaction, based on the outcome of any negative event. The authority figure may be shielded from some of the more adverse effects of that risk, and in fact may even be the *cause* of that event, depending upon the context.

### Similarities and differences to prior meta-analyses on trust

**Figure 9:**
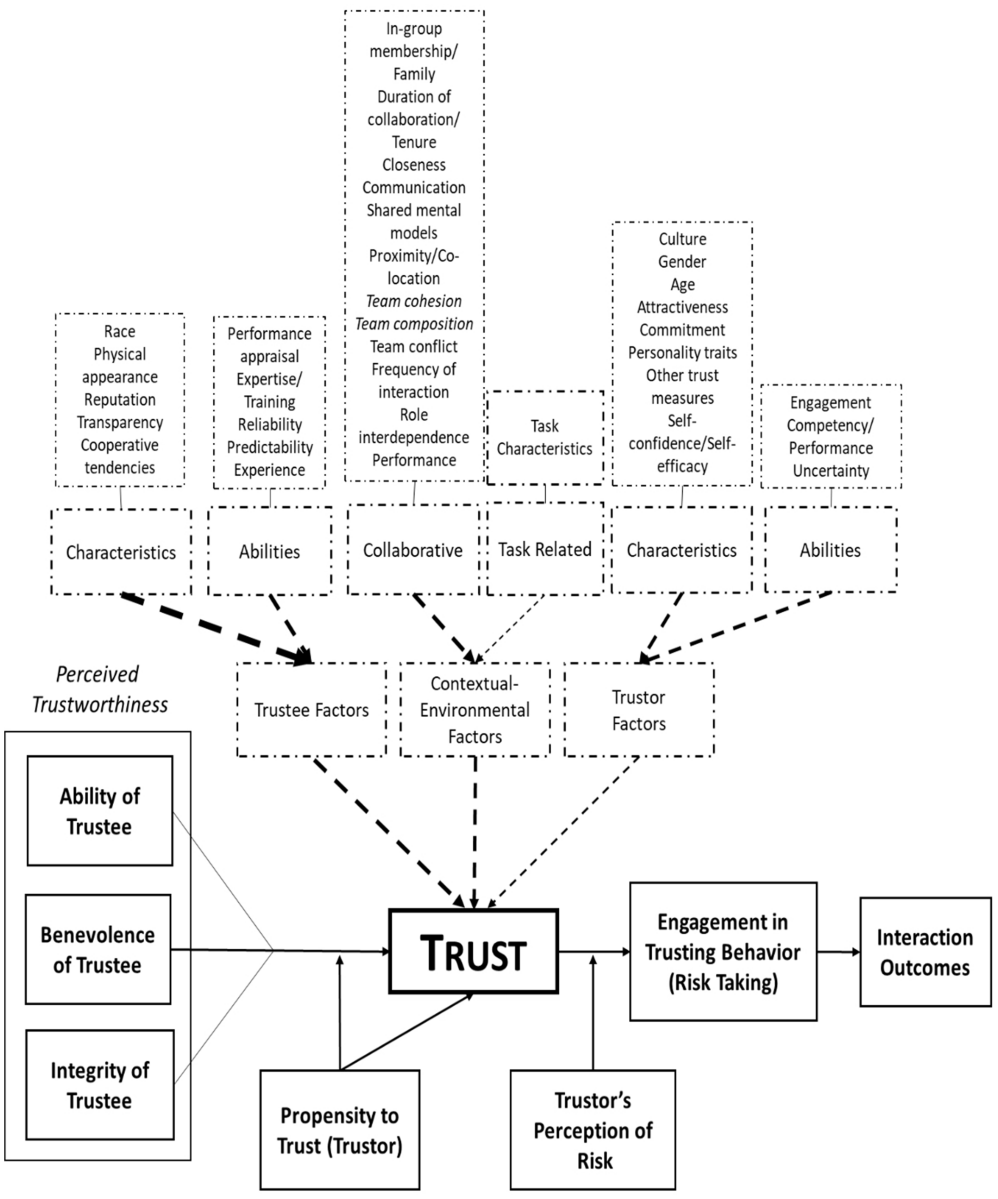
Revised model for factors influencing trust. Model is revised from Mayer et al. (1995). Solid lines and solid boxes demonstrate concepts previously uncovered as factors exerting force on trust between people. Dotted lines and boxes demonstrate those newly uncovered factors which were found to affect trust between people. The width of the dotted lines demonstrates the extent to which the factors have an effect on trust.

One critical step in our journey toward a more comprehensive understanding and model of trust (see Figure 9), is to compare and contrast how results from previous meta-analyses either confirm or contradict what we report here. Examinations concerning the factors that affect trust have been completed in the fields of employee trust in their leaders (Deluga, 1994; Dirks and Ferrin, 2002), customer trust in salespeople (Swan et al., 1999), and how trust is related to risk-taking and job performance (Colquitt et al., 2007). Deluga (1994) reported that supervisor fairness was the largest predictor of subordinates’ trust. Dirks and Ferrin (2002) subsequently found that the behavior, attitude, and support provided by the supervisor all represented strong predictors of trust in that supervisor. We established additional and substantive support for each of these conclusions, as our results demonstrated that a supervisor’s reliability their expertise, their reputation, their performance, and their transparency, were all positively associated with their subordinate’s trust. Additionally, we here established a further supervisor trait that exerts a clear and demonstrable impact on subordinate trust. This is the supervisor’s personality. Our results also addressed shared factors between subordinates and supervisors that affect trust in that supervisor. These effects show that the context of the situated interaction matters, as exchange frequency, communication, interdependence, team performance, risk, shared mental models, and team cohesion all exert significant influences when determining how much trust subordinates place in their supervisor.

One of the major difference from the prior states of understanding that we can now report here concerns effects associated with downward trust. The meta-analyses cited above focused solely on trust in a supervisor or trust that is being directed upward. Our work, however, also examined trust in the subordinate by the supervisors themselves. These results show that performance and reliability of that subordinate exerted influential impacts on a supervisor’s trust in their employees. However, influences of supervisor trust in subordinates also extends to the context of the situation. These effects include communication, team performance, team cohesion, and task type. Based on this information, our present work greatly expands upon the understanding of what influences trust in these working and workplace relationships. In addition to upward trust, a prior analysis has specifically examined customer trust in salespersons (Swan et al., 1999). Swan and colleagues concluded that customer trust in salespeople was generated mostly from the reputation of the firm where that individual was employed. However, it is also somewhat influenced by the behaviors and attributes of the individual attending salesperson themselves. Here, once again, our work has provided strong confirmation that context matters and, critically, that context can be an impactful source of influence in all forms of trust relationship. Our current evaluation, since it was directed to more general coverage, did not focus on the detailed specifics of the relationship between customers and salespeople. However, we were able to expand on the spectrum of these identified contextual factors that influence trust, and so have expanded the overall comprehension of trust’s impact. These included the identification of in-group membership, shared mental models, team cohesion, communication, interdependence, performance, interaction frequency, tenure, team composition, conflict, and task characteristics.

In the final of the prior meta-analysis that we featured and evaluated, Colquitt et al. (2007) investigated how trust is related to risk-taking and job performance. Their findings were again limited in scope, since their criteria for inclusions required that all participants be “*working in a task-focused environment* (p. 912),” and thus, they overwhelmingly examined job performers. Nevertheless, there were similarities between their findings and the wider pattern of results established here. Colquitt and colleagues found the correlational relationship between a trustor’s trust propensity and measured trust to be small but significant (*r =* 0.27). Our more comprehensive findings here confirmed both the size and the direction of these effects (*r =* 0.22). This concordance serves to demonstrate that trust is based more on the immediate situation that is faced as compared to one’s propensity to trust, although, we must affirm that such a propensity still exerts some influence on a trustor’s beliefs. Findings were also similar between the outcomes of Colquitt and associates and our own when the correlation between measured trust and performance was considered. Here the former authors labeled performance as a consequence of trust (*r =* 0.33), while we reported it as an antecedent of trust (*r =* 0.28). Their analysis included the relationships between measured trust and ability (*r =* 0.67), benevolence, (*r =* 0.63), and integrity (*r =* 0.67). These being the three factors that comprise definition of trustworthiness of Mayer et al. (1995). In our analysis, we collapsed these factors into the antecedent of “trustworthiness,” including any measure of ability, benevolence, or integrity. We found a slightly lower, but overall, similar, positive correlation (*r =* 0.47). These values were closer when we examined trustworthiness only of a supervisor, (*r =* 0.56), but, concomitantly, more disparate with the findings of Colquitt and colleagues when we examined trustworthiness of a subordinate (*r =* 0.35). Where these respective analyses most evidently differed was in (i) the sample populations that were include/excluded, and (ii) the number of antecedents of trust that were examined. Our current meta-analysis included many more antecedents than just the propensity to trust or Mayer’s three factors of trustworthiness alone. Additionally, we here approached the issue of risk as something that was shared between the trustor and the trustee, and as an antecedent of trust. Colquitt et al. (2007), examined risk-taking behavior as a consequence of trust. We have proposed here that risk-taking behavior is not a consequence of trust, but that perception of risk is mediated in trust relationships. Our prior reference to trust as “*a calculated exposure to risk*” indicates that, depending on the level of trust, one may well then experience the perceived level of risk differently. That is, would a risky act be seen and comprehended as less so when one’s trustee is one who is highly transparent? Especially in a workplace context, there proves to be a fairly substantive relationship between transparency and trust (*r* = 0.48) for overall trustees. Also, there is a similar effect (*r* = 0.45) for trust in supervisors. If a trustor can already predict the supervisor’s response, it may well be that their perception of risk is, as a consequence greatly attenuated, rather than increasing their risk-taking behavior *per se*.

Where our present pattern of outcomes diverges most evidently from each of the prior identified meta-analyses is in the area of contextual, or shared factors. Interestingly, contextual influences are the only factors that can be manifestly and actively manipulated. The characteristics of the task which is being either individually or mutually shared can be changed. For example, teams and their composition can be altered. Relatively immaleable human characteristics, both on the part of the trustor and the trustee, each exert evident, demonstrable, and measurable impacts on trust. However, regardless of how much an individual understands this fact, they cannot necessarily change such characteristics at will. No team leader can decide that, after they have learnt that trustor propensity to trust has a significant impact on trust in a supervisor, they can simply dial up an increase in their subordinate’s propensity to trust. The contextual factors, that we are here the first to specifically identify, and which evidently do influence trust, can be changed in order to create an environment in which trust flourishes and levels of trust can be titrated and calibrated appropriately.

Despite ours being the first meta-analytic report that provides an overview as to which of these contextual factors influence trust, many organizations appear to be already attempting thereby to increase trust within their own demesnes. For instance, people rely on the reputation of organizations, often *via* social media communications, to decide if they will place their trust in them or not. Hiring companies now do not just review professional sites, they often search for specific candidates’ names online, looking for cues and information as to their trustworthiness. As a result of such personal and corporate searches attempting to assess a person’s trustworthiness, new purpose-directed companies have emerged that are willing to manage online reputations in order to ensure that the most favorable information and interpretations are presented. Anything less than desirable is relegated, by manipulation, into the background of the search. And, of course, on-line scams are all about misplaced trust. We therefore advocate for further research to explore these growing dimensions of interactive trust propensity in order to determine just how each of these constantly changing and evolving contextual factors influences perceived trust.

### Implications of human-human trust findings for human-automation-autonomy interactions

Our present work serves to originate upon, expand, and clarify current understanding in respect of how humans trust each other and the contextual effects which affect those relationships. However, as we have noted, the implications of human-human trust findings extend well beyond human-only relationships. In respect of these prospective lessons, we here explore how conclusions from human-human trust can further apply to trust in developing relationships with automated and autonomous technologies (Billings et al., 2012a,b,c; Hancock et al., 2021a,b). Interest in human trust in other non-human entities (e.g., human-machine trust) is growing dramatically (Sanders et al., 2019). This is especially true since people are beginning to now realize the wider implications and importance of their own personal trust in emerging technologies. The present degree of understanding suggests that humans tend to interact with people and machines in somewhat similar, but not identical ways. Automated decision aids are often designed with characteristics which manifestly and intentionally attempt to emulate human forms of interactions (e.g., human language structures, social etiquette etc.; Madhavan and Wiegmann, 2007). Research here has shown that, although users are clearly conscious that computers are not people, they still often tend to apply human social rules and behaviors to their interactions with them, especially when their conscious awareness of the difference has been blunted by frequent or prolonged interaction. Nass and Moon (2000) have referred to this phenomenon as *ethopoeia*. Essentially this means that we treat something generally in a human fashion even though we remain consciously aware that it does not fully exhibit that kind of social behavior or human attributes (Brave and Nass, 2007; Hancock, 2011). Others have also asserted that it is valuable to make these comparisons between trust in another human and trust in a technological artifact, such as a machine or robot (e.g., Komiak et al., 2005). This attributional propensity is anticipated to become increasingly the case as technology moves from “tool to teammate” (Ososky et al., 2013), even to the extent of extending fundamental attribution errors to nonhuman collaborators (Hancock et al., 2021a,b). Many of the antecedents of trust that already exist for human-human teams bear significant similarities to those that exist in present human-automation teams (Schaefer et al., 2016). Any trustor related factors, or contextual factors can readily be equivalent, regardless of whether the trustee is human or machine. Trustee factors such as transparency, reputation, reliability, predictability, and performance are all factors that can be attributes of machines, as well as humans. As automation at present, typically takes a subservient role, we believe the best current comparison is through use of the present results concerning trustworthiness of a subordinate. It will probably be these data, pro tem, that most usefully serve to forecast which factors will be significant in predicting trust in automation. Trust in ever more autonomous systems is much more liable to be represented by understanding derived from the lateral power relationship structure (and see Hancock, 2017). We, therefore, argue here that the outcomes of our present meta-analysis have both implications for, and direct applications beyond, traditional human relationships. They possess important information for the structuring and functioning of emerging society, more and more dominated by impactful and intention-expressing technology. Understanding the dimensions of human-human trust, as distilled here, may therefore benefit innovators such as roboticists and indeed designers in multiple other fields currently shaping many forms human-machine interaction in order to incorporate this knowledge into their innovative designs.

### Some limitations of the present work and a future roadmap

A number of the issues which presently serve to make comparisons difficult across disparate studies include, but are not limited to: (1) the remaining differences in the definitions of trust which persist across different disciplines (e.g., economics, sociology, politics, psychology, machine intelligence, etc.), (2) the different types and forms of trust that are assessed (e.g., general trust vs. trust in a specific persons or entities), (3) different approaches used to measure trust (e.g., objective performance assessment vs. subjective perception vs. physiological profiles of trust), (4) differing trust referents, (5) differences between trust and trustworthiness, and finally (6) differences in group level vs. individual-level analysis of data. Specific grouping factors are also important but often the experimental evidence that we currently possess does not support a sufficiently reliable set of comparisons. For example, extant literature does not presently permit any definitive analysis of gender differences. This is because too few experimental works have actually reported upon these differences so as to determine how the respective sexes express any significant difference in trust. Of course, this does not mean that such data were not collected but rather that they have been insufficiently reported so as to make any meta-analytic outcome statistically convincing. Additionally, age has, to date, only sporadically been systematically explored in any meaningful fashion. Collectively, this makes it almost impossible to distinguish what effects we have reported are contingent, for example, upon the age or gender of the people involved. As with any meta-analysis, we can only explore the mediators that do provide sufficient information to calculate their influence. Gender, age, race, social class, and other characteristics may exert even greater impact than the ones we have reported but as yet, we cannot confidently determine such impacts. Thus, we recommend that these important moderators deserve and receive more thorough exploration in the coming years.

Despite the broad swath of literature that we did identify concerning trust, we want to emphasize that there are still many forms of human-human trust that remain to be explored. Not only should we be looking more closely at the specific context of trust between people within laboratory environments, but also at natural interactions well beyond these controlled confines. This can be accomplished *via* ethnographic studies embedded in real world experiences. Longitudinal studies of trust evolution are also advisable, specifically in order to gauge how trust initially develops and is then sustained, and/or dissolves across time. As trust is dynamic, any comprehensive model must include time as being perhaps even trust’s most significant modifier. It is critically important then to plot the mutual evolution of the attitudes and dynamic trust responses of both trustor and trustee. This is because it is these emergent, and even momentary levels of relative trust and distrust that may well serve to dictate the nature of recorded interactions. This, as opposed to any singular, absolute level of trust of each of the individuals, or group of individuals involved. Controlled experimental situations need to identify and manipulate these respective, mutual profiles and the way in which past experience and prospective expectations mediate trust expression. As with other forms of behavioral affect (e.g., cognitive workload, Hancock and Matthews, 2019; Hancock G. M. et al., 2021; Longo et al., 2022), prior levels of experience predicate current levels of reaction. In its turn, prospective expectations calibrate current experience, or as the Swan of Avon had it, “*what’s past is prolog*.”

From our present meta-analytic findings, we can certainly provide a much more advanced model which we have illustrated in Figure 9. However, the next step will almost certainly involve a much more sophisticated and dynamic, process-based model that can evaluate, for example, whether a trustor is more likely to trust someone based on the immediate context of their first meeting: a propensity we here term “*insta-trust*.” Subsequently, such a model could account for the influence of each of the other predictors we have identified herein. All studies that we were able to qualify and code, so far, represent point comparisons rather than dynamic and changing profiles of trust that would more veridically reflect how people feel and behave at any moment in time. Important questions which cannot presently be answered by the current assemblage of quantitative data include, how and where is trust broken? Also, precisely how is trust built up in the first place? Is the degree of fracture of trust always proportional to the time it has taken to build up and any levels that it has attained previously? Are there necessarily ceiling and floor effects in trust? These and other crucial dimensions of knowledge cannot be found without an experimental pivot to dynamical measurements and the establishment of prototypical profiles of trust against which to calibrate individual instances.

### Differentiation of trust constructs and their measurement

Although the studies included in our meta-analysis each met our strict inclusion criteria, it may be of some benefit in order to further examine studies on the penumbra of the current acceptance threshold. This action could serve to create a more liberal exposition of the world of trust. This may be beneficial in understanding how diverse researchers, particularly from even more disparate disciplines, ultimately choose their definitions and measures of trust. For example, investigations may claim to be measuring trust, but methods used to assess this construct often prove to be very different (e.g., see Rotter, 1967). Additionally, some researchers refer to trust as a variable in their studies, but they may well actually be referring not to trust *per se*, but to trustworthiness. As we have shown, trust and trustworthiness are actually different constructs that need to be carefully distinguished and measured in different ways. Future work should identify such discrepancies as to how trust-related terminologies are used. Several caveats are therefore required when researching and measuring trust. First, what exactly is being measured? The construct of trust has been parsed into many different sub-categories (and see Hancock et al., 2021a,b). These include, but are not necessarily limited to, affective and cognitive trust (McAllister, 1995), history-based trust (Merritt and Ilgen, 2008), dispositional trust (Merritt and Ilgen, 2008), propensity to trust (Rotter, 1967), and trustworthiness (Mayer et al., 1995; and see the present Table 3). Each sub-category focuses on a slightly different aspect of trust, and therefore, may require different scales and measurement techniques to capture their essence effectively.

Second, when precisely should trust be measured. The empirical findings described here were most frequently based on data that were collected in empirical studies in which trust was measured after the actual interaction had occurred. However, it is important to again emphasize the non-stationarity of trust since ongoing interactions and relational histories continuously influence trust levels at any given point in time. Consequently, trust manifested before, during, or following any particular interaction is almost certainly not equivalent, and past and future trust in the same individual or group will likely change as those relationships evolve (and see Blomqvist, 1997). Obtaining the most reliable and accurate reflection of the changing nature of trust in an interaction may therefore necessitate measuring trust at multiple instances. Future research should continue to address this question as to what the most appropriate junctures to measure trust are. Third, and even more specifically how exactly should trust be measured? A large majority of the research we have here identified used subjective measures. Mostly, this means that a survey was administered asking an individual to rate his/her level of trust in a certain situation, person, or entity. However, these subjective, self-report measures may not directly correspond to the actual behavior, expressed in the observable actions of the individual (see Natsoulas, 1967; Hancock, 1996; Hancock and Matthews, 2019). Complementing subjective scales with other types of measures (e.g., objective behavioral actions; neurophysiological reflections, use choice etc.) is a valuable way of better assessing actually expressed trust in inter-personal interactions (and see Sanders et al., 2019). Some researchers posit that developing a quantitative and objective measure of trust is therefore a necessary precursor to the veridical identification of factors influencing trust in teams, as well as understanding how trust impacts other outcomes (e.g., performance; Adams et al., 2004).

In the realm of economics, measuring interpersonal trust has been accomplished through the use of several objective measures that are frequently employed. These include the Trust Game and the Prisoner’s Dilemma. The Trust Game (Berg et al., 1995) is a behavioral measure of trust that does not create a zero-sum environment (i.e., where one person’s gain necessarily leads to another person’s loss). In the Trust Game, the trustor decides what amount of money to invest in another person (trustee). The money passed to the trustee is tripled, and then the trustee must decide how much to send back to the trustor. Essentially, trust is conceived here as a reflection of the amount of money the trustor invests. Another similar, and nominally objective measure comes *via* the Ultimatum Game. Here, two players are allocated a sum of money. One person proposes how to split the money, and the other person is given the option of whether to accept or reject that proposal. If the offer is rejected, neither person gets anything. The Dictator game is played much like this Ultimatum Game, except that the person must accept any offer proposed (Anderson and Dickinson, 2010). The Prisoner’s Dilemma is one which employs a social conundrum in which two “suspects” are taken into custody and separated. They are both given a choice to support (cooperate) with his/her partner, or to betray them in some fashion (i.e., defect or compete). Choices must be made without knowledge of the choice made by the other person. There are four behavioral outcomes: mutual cooperation, mutual defection, the sucker’s payoff, and the temptation to defect (Axelrod, 1984). The trust an individual has in his/her partner is herein reflected by these four eventualities.^1^ However, caution must still be exercised when using such objective measures. It is critical to verify that the measure is an accurate and real reflection of trust, and not simply reflective of some other associated construct or even previously learned ‘gaming’ strategy. The challenge which currently faces trust research, as it does in a variety of other areas characterized as energetic aspects of human cognition, is precisely how to compare and integrate these differing forms of measure of what is essentially one single emergent state of conscious experience. This challenge is on-going.

## Summary, conclusion, and future challenges

The idea of trust is not without its contemporary critics (Bolton, 2022). However, the present meta-analytic findings demonstrate, that while much is known about trust, there still remains much to learn and this emerging knowledge might well address some contemporary criticisms. Among the latter are a quinary of key concerns. First, we need to establish a much clearer picture concerning the dynamics of both the acute and chronic expressions of trust (Hoffman et al., 2009; Hancock, 2021). This requires a more detailed and articulated account of the onset, rise, sustenance, and dissolution of trust over intervals ranging from moments even to lifetimes. Second, a more detailed exposition is required concerning the reciprocity of trust. While some indicators of this have been proposed and evaluated here, the reasons why trust dyads, triads, etc. develop and persist is still insufficiently articulated. This leads to a third requirement. If the field is to take its next substantive step, more understanding concerning the persisting question of individual differences must be embraced. While our present meta-analytic results have evidently featured nomothetic tendencies, much in terms of variation between individuals continues to be obscure. Such an idiographic approach is an important adjunct to the current picture and requires a strong and consistent empirical attack to articulate fully (and see Hancock et al., 2009). Fourth, there is a fecund but challenging dimension of trust involved in neurophysiological and neuropsychological explorations. We do not wish to imply that such works have yet to be undertaken, for indeed they are on-going. Yet the bridge between this level of understanding and the composite behavioral picture remains to be fully linked. Fifth and finally, we need a much greater emphasis on the positive impacts of trust. When the common element of the definition of the area is one which emphasizes risk and fear of loss or harm, the tendency is to see trust in a somewhat negative light. This is wrong. For, clearly, the act of trusting can render many advantages in social circumstances and if stop-loss were the sole or even primary motivation we ought to ask how, in evolutionary terms, trust has persisted as a trait of so many living organisms. Obviously, the context of trust and those advantages have to be understood, especially as humans are asked, more and more, to trust the technological systems that frame and impact their social world.

In this work, we have reported on the predictors of trust that a summed evaluation of the literature supports, as well as those factors which it currently fails to support. Further, we have identified a number of areas in which results from both correlational and experimental studies are still lacking. As well as a new and more comprehensive model of trust (Figure 9), our present meta-analysis provides the most comprehensive, in-depth evaluation of trust predictors that has been reported to date. In doing so, we pose important questions as to how trust is built, maintained, and then either dissolved, or actively broken. Based on the information uncovered in this analysis, we suggest future research focus on the different dimensions that we have identified which can impact the relationships between people in order to uncover new factors which can be manipulated to positively influence human trust propensities and actual expressions of trust in the world.

## Data availability statement

The original contributions presented in the study are included in the article/Supplementary material, further inquiries can be directed to the corresponding author.

## Author contributions

The research project was led by PH who also wrote the present version of the work, edited a sequence of revisions, and contributed to data analysis and interpretation. TK, AK, and KiS each coded the full panoply of accepted works and then cross-coded same. They each contributed to the writing of the work most especially in editing and revising later versions of the paper. JB provided insights on the interpretation of the summed findings and contributed to manuscript completion with comments and edits on proceeding versions. DB and KrS organized the original assembly of qualified findings and set up the analytic structure. Alongside the authors noted above, they created the format for analysis and each cross-coded the earliest tranche of admissible findings. JS led all meta-analytic procedures and was instrumental in writing the work, most especially in editing and revising the series of versions composing the developing manuscript. All authors contributed to the article and approved the submitted version.

## Funding

This research was supported, in part, by an appointment to the Student Research Participation Program at U.S. Air Force Research Laboratory (AFRL), 711th Human Performance Wing, Human Effectiveness Directorate, Human Centered Intelligence, Surveillance, and Reconnaissance Division, Human Trust and Interaction Branch administered by the Oak Ridge Institute for Science and Education (ORISE) through an interagency agreement between the U.S. Department of Energy and AFRL.

## Conflict of interest

DB was affiliated with Broky Consulting as the founder and sole owner.

The remaining authors declare that the research was conducted in the absence of any commercial or financial relationships that could be construed as a potential conflict of interest.

## Correction note

A correction has been made to this article. Details can be found at: 10.3389/fpsyg.2025.1651358.

## Publisher’s note

All claims expressed in this article are solely those of the authors and do not necessarily represent those of their affiliated organizations, or those of the publisher, the editors and the reviewers. Any product that may be evaluated in this article, or claim that may be made by its manufacturer, is not guaranteed or endorsed by the publisher.

## Author Disclaimer

The opinions contained herein are those of the authors only and should not be construed as those of the Department of Air Force, Department of Army, or US Government.

## Footnotes

^1^ The maxim of unbreakable mutuality being given in the epithet: "be true to your friends though the heaves fall.
