# Correction: How and why humans trust: A meta-analysis and elaborated model

**DOI:** 10.3389/fpsyg.2025.1651358

**Published:** 2025-08-11

**Authors:** P. A. Hancock, Theresa T. Kessler, Alexandra D. Kaplan, Kimberly Stowers, J. Christopher Brill, Deborah R. Billings, Kristin E. Schaefer, James L. Szalma

**Affiliations:** ^1^Department of Psychology and Institute for Simulation and Training, University of Central Florida, Orlando, FL, United States; ^2^Department of Psychology, University of Central Florida, Orlando, FL, United States; ^3^Department of Management, University of Alabama, Tuscaloosa, AL, United States; ^4^United States Air Force Research Laboratory, Wright Patterson Air Force Base, Dayton, NV, United States; ^5^Broky Consulting, LLC, Hillsboro, OR, United States; ^6^DEVCOM Army Research Laboratory, Aberdeen Proving Ground, Adelphi, MD, United States

**Keywords:** trustors, trustees, meta-analysis, trust, dispositional trust

There was a mistake in Figure 9 as published. The incorrect figure was published. The corrected Figure 9 appears below.

**Figure 9:**
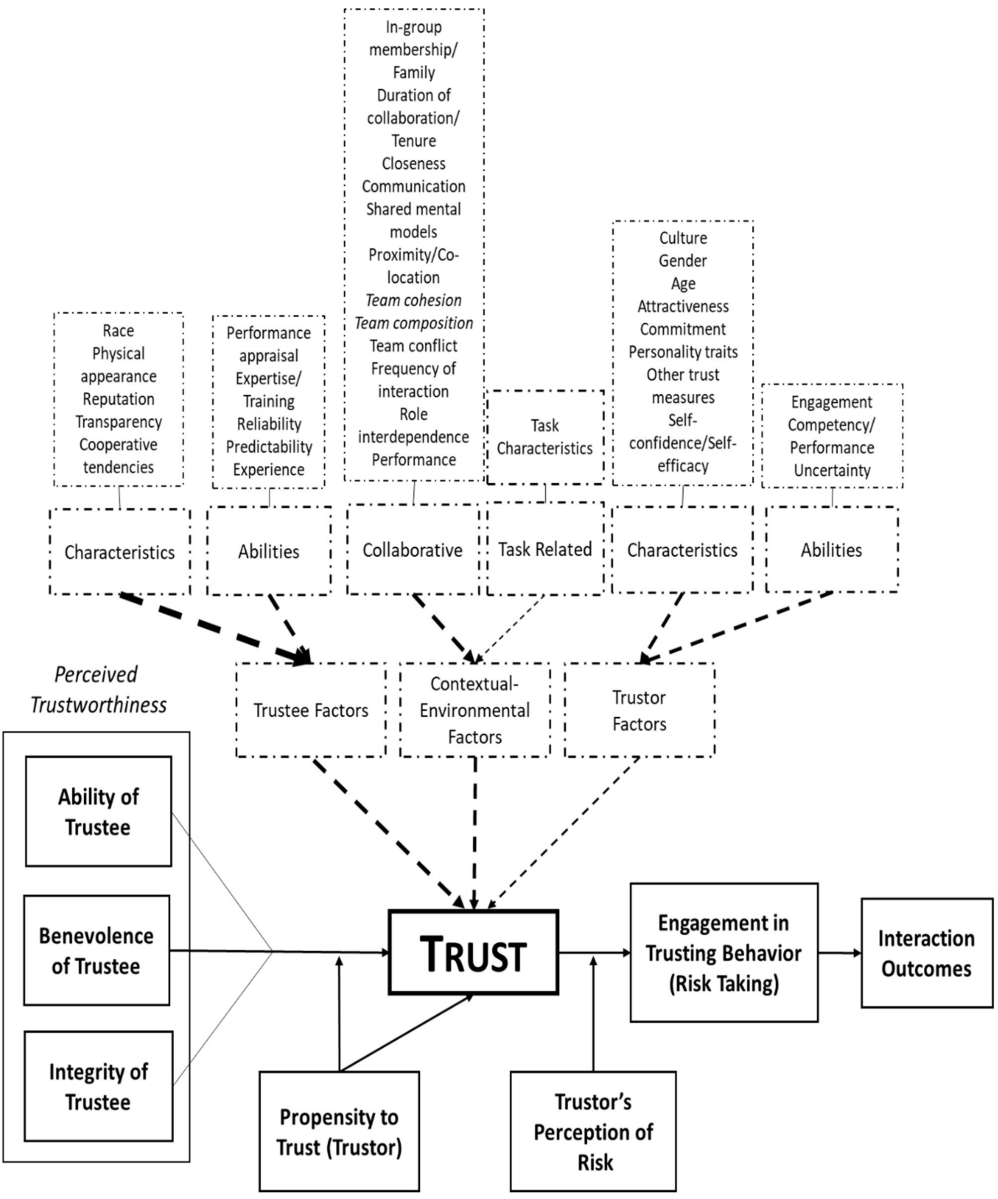
Revised model for factors influencing trust. Model is revised from Mayer et al. (1995). Solid lines and solid boxes demonstrate concepts previously uncovered as factors exerting force on trust between people. Dotted lines and boxes demonstrate those newly uncovered factors which were found to affect trust between people. The width of the dotted lines demonstrates the extent to which the factors have an effect on trust.

Supplemental material Appendix A: Studies Used in this Meta-Analysis was omitted. The Supplemental material file has been updated in the original article.

The original version of this article has been updated.

