# Supplementary material for: How and why humans trust: A meta-analysis and elaborated model
Source: Front Psychol. 2023 Mar 27;14:1081086. doi: 10.3389/fpsyg.2023.1081086 (PMC10083508; doi:10.3389/fpsyg.2023.1081086)
Supplement: Supplementary file 1 [file Table_1.DOCX]

**Appendix A: All Studies Used in the Present Analyses**

Ab Hamid, M.R., Mustafa, Z., Idris, F., Abdullah, M., Suradi, N. R.M., & Ismail, W.R. (2013). Multi-factor of cultural values: a confirmatory factor analytic approach. *Quality & Quantity*, *47* (1), 499-513.

Acar-Burkay, S., Fennis, B.M., & Warlop, L. (2014). Trusting others: the polarization effect of need for closure. *Journal of Personality and Social Psychology*, *107* (4), 719.

Acedo-Carmona, C., & Gomila, A. (2014). Personal trust increases cooperation beyond general trust. *PloS One, 9* (8), e105559.

Adams, C.M., Ware, J.K., Miskell, R.C., & Forsyth, P.B. (2016). Self-regulatory climate: A positive attribute of public schools. *The Journal of Educational Research, 109* (2), 169-180.

Agnihotri, N., Chawla, S., & Jain, P. (2015). Investigating relationship between trust and online communication. *Indian Journal of Health and Wellbeing, 6* (11), 1062-1069.

Agnihotri, R., & Krush, M.T. (2015). Salesperson empathy, ethical behaviors, and sales performance: the moderating role of trust in one's manager. *Journal of Personal Selling & Sales Management, 35* (2), 164-174.

Alamieyeseigha, S. (2012). Likelihood to trust sharing knowledge in multi-cultural consulting companies. *International Journal of Risk and Contingency Management (IJRCM), 1* (2), 16-28.

Alarcon, G.M., Lyons, J. B., & Christensen, J.C. (2016). The effect of propensity to trust and familiarity on perceptions of trustworthiness over time. *Personality and Individual Differences, 94,* 309-315.

Albrecht, S., & Travaglione, A. (2003). Trust in public-sector senior management. *International Journal of Human Resource Management, 14* (1), 76-92.

Allen, J., Jimmieson, N.L., Bordia, P., & Irmer, B E. (2007). Uncertainty during organizational change: Managing perceptions through communication. *Journal of Change Management, 7* (2), 187-210.

Altschuller, S., & Benbunan‐Fich, R. (2010). Trust, performance, and the communication process in ad hoc decision‐making virtual teams. *Journal of Computer‐Mediated Communication, 16* (1), 27-47.

Altuntas, S., & Baykal, U. (2010). Relationship between nurses’ organizational trust levels and their organizational citizenship behaviors. *Journal of Nursing Scholarship, 42* (2), 186-194.

Aly, N.A.E.F.M., & El-Shanawany, S.M. (2016). Nurses’ organizational trust: Its impacts on nurses’ attitudes towards change in critical care and toxicology unites. *International Journal of Information, Business and Management, 8* (4), 205.

Andaleeb, S.S., & Anwar, S.F. (1996). Factors influencing customer trust in salespersons in a developing country. *Journal of International Marketing*, *4* (4), 35-52.

Ariyabuddhiphongs, V., & Kahn, S.I. (2017). Transformational leadership and turnover intention: The mediating effects of trust and job performance on café employees in Thailand. *Journal of Human Resources in Hospitality & Tourism, 16* (2), 215-233.

Arnett, D.B., Wittmann, C.M., & Wilson III, B J. (2004). Encouraging future helping behaviors: The role of student-faculty relationships in higher education marketing. *Journal of Marketing for Higher Education, 13* (1-2), 127-157.

Aryee, S., Budhwar, P.S., & Chen, Z.X. (2002). Trust as a mediator of the relationship between organizational justice and work outcomes: Test of a social exchange model. *Journal of Organizational Behavior, 23* (3), 267-288.

Atuahene-Gima, K., & Li, H. (2002). When does trust matter? Antecedents and contingent effects of supervisee trust on performance in selling new products in China and the United States. *Journal of Marketing, 66* (3), 61-81.

Ayenew, A.A., Gracia, F.J., & Toderi, S. (2015). Linking trust to safety performance in nuclear power plants: The mediating role of team learning. CLEAR *International Journal of Research in Management, Sciences & Technology, 5* (10), 1-14.

Ayoko, O.B., & Pekerti, A.A. (2008). The mediating and moderating effects of conflict and communication openness on workplace trust. *International Journal of Conflict Management, 19* (4), 297-318.

Bailey, P.E., Szczap, P., McLennan, S.N., Slessor, G., Ruffman, T., & Rendell, P.G. (2016). Age-related similarities and differences in first impressions of trustworthiness. *Cognition and Emotion, 30* (5), 1017-1026.

Balkan, M.O., Serin, A.E., & Soran, S. (2014). The relationship between trust, turnover intentions, and emotions: An application. *European Scientific Journal, ESJ*, *10* (2), 73-85.

Ball, G.A., Trevino, L.K., & Sims, H.P. (1993). Justice and organizational punishment: Attitudinal outcomes of disciplinary events. *Social Justice Research, 6* (1), 39-67.

Bammens, Y., & Collewaert, V. (2014). Trust between entrepreneurs and angel investors: Exploring positive and negative implications for venture performance assessments. *Journal of Management, 40* (7), 1980-2008.

Banai, M., Stefanidis, A., Shetach, A., & Özbek, M.F. (2014). Attitudes toward ethically questionable negotiation tactics: A two-country study. *Journal of Business Ethics, 123* (4), 669-685.

Barczak, G., Lassk, F., & Mulki, J. (2010). Antecedents of team creativity: An examination of team emotional intelligence, team trust and collaborative culture. *Creativity and Innovation Management, 19* (4), 332-345.

Bartram, T., & Casimir, G. (2007). The relationship between leadership and follower in-role performance and satisfaction with the leader: the mediating effects of empowerment and trust in the leader. *Leadership & Organization Development Journal, 28* (1), 4-19.

Becerra, M., & Gupta, A.K. (2003). Perceived trustworthiness within the organization: The moderating impact of communication frequency on trustor and trustee effects. *Organization science, 14* (1), 32-44.

Bekk, M., & Spörrle, M. (2010). The influence of perceived personality characteristics on positive attitude towards and suitability of a celebrity as a marketing campaign endorser. *The Open Psychology Journal, 3* (1), 54-66.

Bente, G., Dratsch, T., Kaspar, K., Häßler, T., Bungard, O., & Al-Issa, A. (2014). Cultures of trust: effects of avatar faces and reputation scores on German and Arab players in an online trust-game*. PloS one, 9* (6), e98297.

Bergman, J.Z., Small, E.E., Bergman, S.M., & Rentsch, J.R. (2010). Asymmetry in perceptions of trustworthiness: It’s not you; it’s me. *Negotiation and Conflict Management Research, 3* (4), 379-399.

Bernerth, J.B., & Walker, H J. (2009). Propensity to trust and the impact on social exchange: An empirical investigation. *Journal of Leadership & Organizational Studies, 15* (3), 217-226.

Bianchi, E.C., & Brockner, J. (2012). In the eyes of the beholder? The role of dispositional trust in judgments of procedural and interactional fairness. Organizational Behavior and Human Decision Processes, 118(1), 46-59.

Bierly, P.E., Stark, E.M., & Kessler, E.H. (2009). The moderating effects of virtuality on the antecedents and outcome of NPD team trust. *Journal of Product Innovation Management, 26* (5), 551-565.

Bird, J.J., Wang, C., Watson, J., & Murray, L. (2012). Teacher and principal perceptions of authentic leadership: *Implications for trust, engagement, and intention to return. Journal of School Leadership, 22* (3), 425-461.

Birkenmeier, B.J., & Sanséau, P Y. (2016). The relationships between perceptions of supervisor, trust in supervisor and job performance: A study in the banking industry. *Journal of Applied Business Research, 32* (1), 161.

Boateng, J., & Cox III, R.W. (2016). Exploring the trust question in the midst of public management reforms. *Public Personnel Management, 45* (3), 239-263.

Boies, K., Fiset, J., & Gill, H. (2015). Communication and trust are key: Unlocking the relationship between leadership and team performance and creativity. *The Leadership Quarterly, 26* (6), 1080-1094.

Boies, K., Lvina, E., & Martens, M.L. (2011). Shared leadership and team performance in a business strategy simulation. *Journal of Personnel Psychology. 9,* 195-202.

Brahm, T., & Kunze, F. (2012). The role of trust climate in virtual teams. *Journal of Managerial Psychology, 27* (6), 595-614.

Braun, S., Peus, C., Weisweiler, S., & Frey, D. (2013). Transformational leadership, job satisfaction, and team performance: A multilevel mediation model of trust. *The Leadership Quarterly, 24* (1), 270-283.

Braun, S., Peus, C., Weisweiler, S., & Frey, D. (2013). Transformational leadership, job satisfaction, and team performance: A multilevel mediation model of trust. *The Leadership Quarterly, 24* (1), 270-283.

Brower, H.H., Lester, S. W., Korsgaard, M.A., & Dineen, B.R. (2009). A closer look at trust between managers and subordinates: Understanding the effects of both trusting and being trusted on subordinate outcomes. *Journal of Management, 35* (2), 327-347.

Brger, J., Luke, M., & Indelov, H. (2007). Interpersonal trust in German-Czech work relations: Mutual expectations and suggestions for improvement. *Journal of Organizational Transformation & Social Change, 3* (2), 173-199.

Burns, C., Mearns, K., & McGeorge, P. (2006). Explicit and implicit trust within safety culture. *Risk Analysis, 26* (5), 1139-1150.

Buvik, M.P., & Tvedt, S. D. (2016). The impact of commitment and climate strength on the relationship between trust and performance in cross-functional project teams: A moderated mediation analysis. *Team Performance Management, 22* (3/4), 114-138.

Camelo-Ordaz, C., García-Cruz, J., & Sousa-Ginel, E. (2014). Antecedents of relationship conflict in top management teams. International *Journal of Conflict Management, 25* (2), 124-147.

Cameron, A.F., & Webster, J. (2011). Relational outcomes of multi-communicating: Integrating incivility and social exchange perspectives. *Organization Science, 22* (3), 754-771.

Campbell, D.J., & Campbell, K.M. (2009). Embracing change: Further examination of a “capabilities and benevolence” beliefs model in a military sample. *Military Psychology, 21* (3), 351.

Carter, M.Z., & Mossholder, K.W. (2015). Are we on the same page? The performance effects of congruence between supervisor and group trust. *Journal of Applied Psychology, 100* (5), 1349.

Cassidy, B.S., & Gutchess, A.H. (2015). Influences of appearance-behaviour congruity on memory and social judgements. *Memory, 23* (7), 1039-1055.

Caza, A., Zhang, G., Wang, L., & Bai, Y. (2015). How do you really feel? Effect of leaders' perceived emotional sincerity on followers' trust. *The Leadership Quarterly, 26* (4), 518-531.

Chan, C.H.S., & Mak, W.M. (2014). The impact of servant leadership and subordinates' organizational tenure on trust in leader and attitudes. *Personnel Review, 43* (2), 272-287.

Chou, L.F., Wang, A.C., Wang, T.Y., Huang, M.P., & Cheng, B.S. (2008). Shared work values and team member effectiveness: The mediation of trustfulness and trustworthiness. *Human Relations, 61* (12), 1713-1742.

Chow, P.T., Cheung, S.O., & Ka Wa, Y. (2014). Impact of trust and satisfaction on the commitment-withdrawal relationship. *Journal of Management in Engineering, 31* (5), 04014087-1- 04014087-8.

Christie, A.M., Jordan, P.J., & Troth, A.C. (2015). Trust antecedents: emotional intelligence and perceptions of others. *International Journal of Organizational Analysis, 23* (1), 89-101.

Chughtai, A.A., & Buckley, F. (2013). Exploring the impact of trust on research scientists' work engagement: Evidence from Irish science research centers. *Personnel Review, 42* (4), 396-421.

Chughtai, A.A., & Buckley, F. (2011). Work engagement: antecedents, the mediating role of learning goal orientation and job performance. *Career Development International, 16* (7), 684-705.

Chughtai, A.A., & Buckley, F. (2009). Linking trust in the principal to school outcomes: The mediating role of organizational identification and work engagement. *International Journal of Educational Management, 23* (7), 574-589.

Colquitt, J.A., & Rodell, J.B. (2011). Justice, trust, and trustworthiness: A longitudinal analysis integrating three theoretical perspectives. *Academy of Management Journal, 54* (6), 1183-1206.

Costa, A.C., & Anderson, N. (2011). Measuring trust in teams: Development and validation of a multifaceted measure of formative and reflective indicators of team trust. *European Journal of Work and Organizational Psychology, 20* (1), 119-154.

Costa, A.C., Bijlsma-Frankema, K., & de Jong, B. (2009). The role of social capital on trust development and dynamics: implications for cooperation, monitoring and team performance. *Social Science Information, 48* (2), 199-228.

Costa, A.C., Roe, R.A., & Taillieu, T. (2001). Trust within teams: The relation with performance effectiveness. *European Journal of Work and Organizational Psychology, 10* (3), 225-244.

Costigan, R.D., Insinga, R.C., Kranas, G., Kureshov, V.A., & Ilter, S.S. (2004). Predictors of employee trust of their CEO: A three-country study. *Journal of Managerial Issues*, *16* (2), 197-216.

Coyle‐Shapiro, J.A.M., Morrow, P.C., Richardson, R., & Dunn, S. R. (2002). Using profit sharing to enhance employee attitudes: A longitudinal examination of the effects on trust and commitment. *Human Resource Management, 41* (4), 423-439.

Crisp, C.B., & Jarvenpaa, S.L. (2013). Swift trust in global virtual teams. *Journal of Personnel Psychology.12*, 45-56.

Curseu, P.L., & Otoiu, C. (2013). It is also a matter of time: A systemic and temporal account for the interplay of trust and psychological safety in groups. *Psihologia Sociala*, (32), 169-183.

Daly, A.J., Moolenaar, N.M., Liou, Y.H., Tuytens, M., & Del Fresno, M. (2015). Why so difficult? Exploring negative relationships between educational leaders: The role of trust, climate, and efficacy. *American Journal of Education, 122* (1), 1-38.

Davis, D.D., & Bryant, J.L. (2010). Leader-member exchange, trust, and performance in national science foundation industry/university cooperative research centers. *The Journal of Technology Transfer, 35* (5), 511-526.

De Jong, B.A., & Elfring, T. (2010). How does trust affect the performance of ongoing teams? The mediating role of reflexivity, monitoring, and effort. *Academy of Management Journal, 53* (3), 535-549.

De Jong, B.A., Bijlsma-Frankema, K.M., & Cardinal, L.B. (2014). Stronger than the sum of its parts? The performance implications of peer control combinations in teams. *Organization Science, 25* (6), 1703-1721.

De Jong, B.A., & Dirks, K.T. (2012). Beyond shared perceptions of trust and monitoring in teams: implications of asymmetry and dissensus. *Journal of Applied Psychology, 97* (2), 391.

Demir, B., & Kumkale, G.T. (2013). Individual differences in willingness to become an organ donor: A decision tree approach to reasoned action. *Personality and Individual Differences, 55* (1), 63-69.

DeOrtentiis, P.S., Summers, J.K., Ammeter, A.P., Douglas, C., & Ferris, G.R. (2013). Cohesion and satisfaction as mediators of the team trust–team effectiveness relationship. *Career Development International, 18* (5), 521-543.

Dijk, C., Koenig, B., Ketelaar, T., & de Jong, P.J. (2011). Saved by the blush: being trusted despite defecting. *Emotion, 11* (2), 313.

Dobransky, N.D., & Frymier, A.B. (2004). Developing teacher‐student relationships through out of class communication. *Communication Quarterly, 52* (3), 211-223.

Douglas, C., & Zivnuska, S. (2008). Developing trust in leaders: An antecedent of firm performance. SAM *Advanced Management Journal, 73* (1), 20-28.

Downey, S.N., Werff, L., Thomas, K. M., & Plaut, V.C. (2015). The role of diversity practices and inclusion in promoting trust and employee engagement. *Journal of Applied Social Psychology, 45* (1), 35-44.

Drescher, M.A., Korsgaard, M.A., Welpe, I.M., Picot, A., & Wigand, R.T. (2014). The dynamics of shared leadership: Building trust and enhancing performance. *Journal of Applied Psychology, 99* (5), 771-784.

Eatough, E., Chang, C.H., & Hall, N. (2015). Getting roped in: Group cohesion, trust, and efficacy following a ropes course intervention. *Performance Improvement Quarterly, 28* (2), 65-89.

Ellonen, R., Blomqvist, K., & Puumalainen, K. (2008). The role of trust in organizational innovativeness. *European Journal of Innovation Management, 11* (2), 160-181.

Ertürk, A. (2008). A trust-based approach to promote employees' openness to organizational change in Turkey. *International Journal of Manpower, 29* (5), 462-483.

Etang, A., Fielding, D., & Knowles, S. (2012). Are survey measures of trust correlated with experimental trust? Evidence from Cameroon. *The Journal of Development Studies, 48* (12), 1813-1827.

Evans, A.M., & Revelle, W. (2008). Survey and behavioral measurements of interpersonal trust. *Journal of Research in Personality, 42* (6), 1585-1593.

Ferguson, A.J., & Peterson, R.S. (2015). Sinking slowly: Diversity in propensity to trust predicts downward trust spirals in small groups. *Journal of Applied Psychology, 100* (4), 1012-1024.

Ferreira Peralta, C., & Francisca Saldanha, M. (2014). Knowledge-centered culture and knowledge sharing: the moderator role of trust propensity. *Journal of Knowledge Management, 18* (3), 538-550.

Ferrin, D.L., Bligh, M.C., & Kohles, J.C. (2008). It takes two to tango: An interdependence analysis of the spiraling of perceived trustworthiness and cooperation in interpersonal and intergroup relationships. *Organizational Behavior and Human Decision Processes, 107* (2), 161-178.

Ferrin, D.L., & Dirks, K.T. (2003). The use of rewards to increase and decrease trust: Mediating processes and differential effects. *Organization Science, 14* (1), 18-31.

Finkenauer, C., Kerkhof, P., Righetti, F., & Branje, S. (2009). Living together apart: Perceived concealment as a signal of exclusion in marital relationships. *Personality and Social Psychology Bulletin, 35* (10), 1410-1422.

Flaherty, K.E., & Pappas, J.M. (2009). Expanding the sales professional's role: A strategic re-orientation?. *Industrial Marketing Management, 38* (7), 806-813.

Fleig-Palmer, M.M., Rathert, C., & Porter, T.H. (2018). Building trust: The influence of mentoring behaviors on perceptions of health care managers’ trustworthiness. *Health Care Management Review, 43* (1), 69-78.

Flowe, H.D. (2012). Do characteristics of faces that convey trustworthiness and dominance underlie perceptions of criminality? *PLoS One, 7* (6), e37253.

Fong, P.S., & Lung, B.W. (2007). Interorganizational teamwork in the construction industry. *Journal of Construction Engineering and Management, 133* (2), 157-168.

Fouragnan, E., Chierchia, G., Greiner, S., Neveu, R., Avesani, P., & Coricelli, G. (2013). Reputational priors magnify striatal responses to violations of trust. *Journal of Neuroscience, 33* (8), 3602-3611.

Franklin Jr, R.G., & Zebrowitz, L.A. (2016). The influence of political candidates’ facial appearance on older and younger adults’ voting choices and actual electoral success. *Cogent Psychology, 3* (1), 1151602.

Frazier, M.L., Johnson, P.D., Gavin, M., Gooty, J., & Bradley Snow, D. (2010). Organizational justice, trustworthiness, and trust: A multi-foci examination. *Group & Organization Management, 35* (1), 39-76.

Frazier, M.L., Gooty, J., Little, L.M., & Nelson, D.L. (2015). Employee attachment: Implications for supervisor trustworthiness and trust. *Journal of Business and Psychology, 30* (2), 373-386.

Frieder, R.E., & Basik, K.J. (2017). Political Skill, Behavioral Integrity, and Work Outcomes: Test of a Multistage Model. *Journal of Leadership & Organizational Studies, 24* (1), 65-82.

Fruhen, L.S., Watkins, C.D., & Jones, B.C. (2015). Perceptions of facial dominance, trustworthiness and attractiveness predict managerial pay awards in experimental tasks. *The Leadership Quarterly, 26* (6), 1005-1016.

Fujimura, T., & Okanoya, K. (2016). Untrustworthiness inhibits congruent facial reactions to happy faces. *Biological Psychology, 121*, 30-38.

Fulk, J., Brief, A.P., & Barr, S.H. (1985). Trust-in-supervisor and perceived fairness and accuracy of performance evaluations. *Journal of Business Research, 13* (4), 301-313.

Fulmer, C.A., & Ostroff, C. (2017). Trust in direct leaders and top leaders: A trickle-up model. *Journal of Applied Psychology, 102* (4), 648-657.

Furumo, K., & Pearson, J.M. (2007). Gender-based communication styles, trust, and satisfaction in virtual teams. Journal of Information, *Information Technology & Organizations, 2*, 47-60.

Gbadamosi, G., Ndaba, J., & Oni, F. (2007). Predicting charlatan behaviour in a non-Western setting: lack of trust or absence of commitment? *Journal of Management Development, 26* (8), 753-769.

Gill, H., Boies, K., Finegan, J.E., & McNally, J. (2005). Antecedents of trust: Establishing a boundary condition for the relation between propensity to trust and intention to trust. *Journal of Business and Psychology, 19* (3), 287-302.

Goebel, S., & Weißenberger, B.E. (2017). The relationship between informal controls, ethical work climates, and organizational performance. *Journal of Business Ethics, 141* (3), 505-528.

Gong, Y., Kim, T.Y., Lee, D.R., & Zhu, J. (2013). A multilevel model of team goal orientation, information exchange, and creativity. *Academy of Management Journal, 56* (3), 827-851.

Grand, J.A., Lloyd, J.W., Ilgen, D.R., Abood, S., & Sonea, I.M. (2013). A measure of and predictors for veterinarian trust developed with veterinary students in a simulated companion animal practice. *Journal of the American Veterinary Medical Association, 242* (3), 322-334.

Gregory, J.B., & Levy, P.E. (2011). It's not me, it's you: A multilevel examination of variables that impact employee coaching relationships. *Consulting Psychology Journal: Practice and Research, 63* (2), 67-88.

Greitemeyer, T., & Cox, C. (2013). There's no “I” in team: Effects of cooperative video games on cooperative behavior. *European Journal of Social Psychology, 43* (3), 224-228.

Guenzi, P., De Luca, L.M., & Spiro, R. (2016). The combined effect of customer perceptions about a salesperson’s adaptive selling and selling orientation on customer trust in the salesperson: a contingency perspective. *Journal of Business & Industrial Marketing, 31* (4), 553-564.

Gunia, B.C., Brett, J.M., Nandkeolyar, A.K., & Kamdar, D. (2011). Paying a price: culture, trust, and negotiation consequences. *Journal of Applied Psychology, 96* (4), 774.

Gupta, R. (2015). Understanding clients’ ties to a tax practitioner: the mediating influence of trust and service satisfaction. *Australian Tax Forum.* *30* (2), 357-384.

Haines, R. (2014). Group development in virtual teams: An experimental reexamination. *Computers in Human Behavior, 39*, 213-222.

Hansen, S.D., Dunford, B.B., Alge, B.J., & Jackson, C.L. (2016). Corporate social responsibility, ethical leadership, and trust propensity: A multi-experience model of perceived ethical climate*. Journal of Business Ethics, 137* (4), 649-662.

Hall, D.L., Cohen, A., Meyer, K.K., Varley, A.H., & Brewer, G.A. (2015). Costly signaling increases trust, even across religious affiliations. *Psychological Science, 26*(9), 1368-1376.

Han, G., & Harms, P.D. (2010). Team identification, trust, and conflict: A mediation model. International *Journal of Conflict Management, 21* (1), 20-43.

Hansen, M.H., Morrow Jr, J.L., & Batista, J.C. (2002). The impact of trust on cooperative membership retention, performance, and satisfaction: an exploratory study. *The International Food and Agribusiness Management Review, 5* (1), 41-59.

Harrington, S.J., & Ruppel, C.P. (1999). Telecommuting: A test of trust, competing values, and relative advantage. *IEEE Transactions on Professional Communication, 42* (4), 223-239.

Hasel, M.C. (2013). A question of context: the influence of trust on leadership effectiveness during crisis. *Management, 16* (3), 264-293.

Hassan, M., Nadeem, A.B., & Akhter, A. (2016). Impact of workplace spirituality on job satisfaction: Mediating effect of trust. *Cogent Business & Management, 3* (1), 1189808.

Haynie, J.J., Mossholder, K.W., & Harris, S.G. (2016). Justice and job engagement: The role of senior management trust. *Journal of Organizational Behavior, 37* (6), 889-910.

Hempel, P.S., Zhang, Z.X., & Tjosvold, D. (2009). Conflict management between and within teams for trusting relationships and performance in China. *Journal of Organizational Behavior, 30* (1), 41-65.

Henderson, L.S., Stackman, R.W., & Lindekilde, R. (2016). The centrality of communication norm alignment, role clarity, and trust in global project teams. *International Journal of Project Management, 34* (8), 1717-1730.

Hiraishi, K., Yamagata, S., Shikishima, C., & Ando, J. (2008). Maintenance of genetic variation in personality through control of mental mechanisms: A test of trust, extraversion, and agreeableness. *Evolution and Human Behavior, 29* (2), 79-85.

Hofhuis, J., van der Rijt, P.G., & Vlug, M. (2016). Diversity climate enhances work outcomes through trust and openness in workgroup communication. *Springer Plus, 5* (1), 714-728.

Holland, P., Cooper, B., & Sheehan, C. (2017). Employee voice, supervisor support, and engagement: The mediating role of trust. *Human Resource Management, 56* (6), 915-929.

Holtz, B.C. (2015). From first impression to fairness perception: Investigating the impact of initial trustworthiness beliefs. *Personnel Psychology, 68* (3), 499-546.

Hoy, W.K. (1992). Faculty Trust in Colleagues: Linking the Principal with School Effectiveness. *Journal of Research and Development in Education, 26* (1), 38-45.

Hsieh, C.C., & Wang, D.S. (2015). Does supervisor-perceived authentic leadership influence employee work engagement through employee-perceived authentic leadership and employee trust? The International *Journal of Human Resource Management, 26* (18), 2329-2348.

Hsu, M.H., & Chang, C.M. (2014). Examining interpersonal trust as a facilitator and uncertainty as an inhibitor of intra‐organizational knowledge sharing. *Information Systems Journal, 24* (2), 119-142.

Huang, J. . (2012). Be proactive as empowered? The role of trust in one's supervisor in psychological empowerment, feedback seeking, and job performance. *Journal of Applied Social Psychology, 42*, E103-E127.

Huang, X., Iun, J., Liu, A., & Gong, Y. (2010). Does participative leadership enhance work performance by inducing empowerment or trust? The differential effects on managerial and non‐managerial subordinates. *Journal of Organizational Behavior, 31* (1), 122-143.

Hubbell, A.P., & Chory‐Assad, R.M. (2005). Motivating factors: Perceptions of justice and their relationship with managerial and organizational trust. *Communication Studies, 56* (1), 47-70.

Indartono, S., & Chen, C.H.V. (2011). Moderating effects of tenure and gender on the relationship between perception of organizational politics and commitment and trust. *South Asian Journal of Management, 18* (1), 7-36.

Isik, M., & Aliyev, Y. (2015). The relationship between teamwork and organizational trust. *International* *Journal of Research in Business and Social Science,* *4* (1), 113-132.

Ivaskovic, I. (2014). The effect of HRM quality on trust and team cohesion*. Economic and Business Review for Central and South-Eastern Europe, 16* (3), 337-365,371-372.

Jang, C.Y. (2013). Facilitating trust in virtual teams: The role of awareness. *Journal of Competitiveness Studies, 21* (1/2), 61.

Jonasson, C., Normann, J., & Jakob, L. (2014). Faculty trust, conflict and the use of knowledge in an international higher education context. *Journal of Educational Sciences and Psychology, 4* (2), 1-15.

Johansen, S.T., Selart, M., & Grønhaug, K. (2013). The effects of risk on initial trust formation. *Journal of Applied Social Psychology, 43* (6), 1185-1199.

John, N.A., Seme, A., Roro, M.A., & Tsui, A.O. (2017). Understanding the meaning of marital relationship quality among couples in peri-urban Ethiopia. *Culture, Health & Sexuality, 19*(2), 267-278.

Johnson, D., & Grayson, K. (2005). Cognitive and affective trust in service relationships. *Journal of Business Research, 58* (4), 500-507.

Johnson, H., Worthington, R., Gredecki, N., & Wilks-Riley, F.R. (2016). The relationship between trust in work colleagues, impact of boundary violations and burnout among staff within a forensic psychiatric service. *Journal of Forensic Practice, 18* (1), 64-75.

Jung, D.I., & Avolio, B.J. (2000). Opening the black box: An experimental investigation of the mediating effects of trust and value congruence on transformational and transactional leadership. *Journal of Organizational Behavior*, *21* (8), 949-964.

Kang, I., Jeon, S., Lee, S., & Lee, C.K. (2005). Investigating structural relations affecting the effectiveness of service management. *Tourism Management, 26* (3), 301-310.

Kenworthy, J.B., & Jones, J. (2009). The roles of group importance and anxiety in predicting depersonalized ingroup trust. *Group Processes & Intergroup Relations, 12* (2), 227-239.

Khan, M.S., Breitenecker, R J., Gustafsson, V., & Schwarz, E.J. (2015). Innovative entrepreneurial teams: The give and take of trust and conflict. *Creativity and Innovation Management, 24* (4), 558-573.

Khuong, M.N. (2015). The Effect of Ethical Leadership and Organizational Justice on Employee Engagement--The Mediating Role of Employee Trust. *International Journal of Trade, Economics and Finance, 6* (4), 235-240.

Kidron, A., Tzafrir, S.S., & Meshoulam, I. (2016). All we need is trust: trust and human resource management. *Team Performance Management, 22* (3/4), 139-155.

Kim, T.Y., Lee, D.R., & Wong, N.Y.S. (2016). Supervisor humor and employee outcomes: The role of social distance and affective trust in supervisor. *Journal of Business and Psychology, 31* (1), 125-139.

Kim, T.Y., Wang, J., & Chen, J. (2018). Mutual trust between leader and subordinate and employee outcomes. *Journal of Business Ethics, 149* (4), 945-958.

Kong, D.T. (2015). Narcissists’ negative perception of their counterpart’s competence and benevolence and their own reduced trust in a negotiation context. *Personality and Individual Differences, 74*, 196-201.

Krosgaard, M.A., Brodt, S.E., & Whitener, E.M. (2002). Trust in the face of conflict: The role of managerial trustworthy behavior and organizational context. *Journal of Applied Psychology, 87* (2), 312-319.

Kougiannou, K., Redman, T., & Dietz, G. (2015). The outcomes of works councils: the role of trust, justice, and industrial relations climate. *Human Resource Management Journal, 25* (4), 458-477.

Krasman, J. (2014). Do my staff trust me? The influence of organizational structure on subordinate perceptions of supervisor trustworthiness. *Leadership & Organization Development Journal, 35* (5), 470-488.

Ladebo, O.J. (2006). Perceptions of trust and employees’ attitudes: A look at Nigeria’s agricultural extension workers. *Journal of Business and Psychology, 20* (3), 409-427.

Langfred, C.W. (2007). The downside of self-management: A longitudinal study of the effects of conflict on trust, autonomy, and task interdependence in self-managing teams. *Academy of Management Journal, 50* (4), 885-900.

Langfred, C.W. (2004). Too much of a good thing? Negative effects of high trust and individual autonomy in self-managing teams. *Academy of Management Journal, 47* (3), 385-399.

Lapointe, É., Vandenberghe, C., & Boudrias, J.S. (2014). Organizational socialization tactics and newcomer adjustment: The mediating role of role clarity and affect‐based trust relationships. *Journal of Occupational and Organizational Psychology, 87* (3), 599-624.

Laschinger, H.K.S., Leiter, M.P., Day, A., Gilin-Oore, D., & Mackinnon, S.P. (2012). Building empowering work environments that foster civility and organizational trust: Testing an intervention. *Nursing Research, 61* (5), 316-325.

Lau, D.C., Lam, L.W., & Salamon, S.D. (2008). The impact of relational demographics on perceived managerial trustworthiness: Similarity or norms.? *The Journal of Social Psychology, 148* (2), 187-209.

Lau, D.C., Lam, L.W., & Wen, S.S. (2014). Examining the effects of feeling trusted by supervisors in the workplace: A self‐evaluative perspective. *Journal of Organizational Behavior, 35* (1), 112-127.

Lau, D.C., & Liden, R.C. (2008). Antecedents of coworker trust: Leaders' blessings. *Journal of Applied Psychology, 93* (5), 1130-1138.

Lauring, J., & Selmer, J. (2012). Openness to diversity, trust, and conflict in multicultural organizations. *Journal of Management & Organization, 18* (6), 795-806.

Lee, P., Gillespie, N., Mann, L., & Wearing, A. (2010). Leadership and trust: Their effect on knowledge sharing and team performance. *Management Learning, 41* (4), 473-491.

Lee, S.M., Lim, K.J., Swanson, E., Park, D.H., & Lee, Y.K. (2016). Authentic Leadership and its Consequences in a Hotel Restaurant Context. *Global Business and Finance Review, 21*, 1-19.

Lehmann‐Willenbrock, N., Lei, Z., & Kauffeld, S. (2012). Appreciating age diversity and German nurse well‐being and commitment: Co‐worker trust as the mediator. *Nursing & Health Sciences, 14* (2), 213-220.

Levin, D.Z., & Cross, R. (2004). The strength of weak ties you can trust: The mediating role of trust in effective knowledge transfer. *Management Science, 50* (11), 1477-1490.

Levin, D.Z., Whitener, E.M., & Cross, R. (2006). Perceived trustworthiness of knowledge sources: The moderating impact of relationship length. *Journal of Applied Psychology, 91* (5), 1163-1171.

Li, A.N., & Tan, H.H. (2013). What happens when you trust your supervisor? Mediators of individual performance in trust relationships. *Journal of Organizational Behavior, 34* (3), 407-425.

Li, N., Yan, J., & Jin, M. (2007). How does organizational trust benefit work performance?. *Frontiers of Business Research in China, 1* (4), 622-637.

Li, S. L., Huo, Y., & Long, L.R. (2017). Chinese traditionality matters: Effects of differentiated empowering leadership on followers’ trust in leaders and work outcomes. *Journal of Business Ethics, 145* (1), 81-93.

Lin, T.C., & Huang, C.C. (2009). Understanding social loafing in knowledge contribution from the perspectives of justice and trust. *Expert Systems with Applications, 36* (3), 6156-6163.

Lin, T.C., & Huang, C.C. (2009). Understanding the determinants of EKR usage from social, technological, and personal perspectives. *Journal of Information Science, 35* (2), 165-179.

Lines, R., Selart, M., Espedal, B., & Johansen, S.T. (2005). The production of trust during organizational change. *Journal of Change Management, 5* (2), 221-245.

Liu, J., Siu, O. L., & Shi, K. (2010). Transformational leadership and employee well‐being: The mediating role of trust in the leader and self‐efficacy. *Applied Psychology, 59* (3), 454-479.

Liu, J., Rau, P.L.P., & Wendler, N. (2015). Trust and online information-sharing in close relationships: a cross-cultural perspective. *Behaviour & Information Technology, 34* (4), 363-374.

Liu, M., & Wang, C. (2010). Explaining the influence of anger and compassion on negotiators’ interaction goals: An assessment of trust and distrust as two distinct mediators. *Communication Research, 37* (4), 443-472.

Madjar, N., & Ortiz-Walters, R. (2009). Trust in supervisors and trust in customers: Their independent, relative, and joint effects on employee performance and creativity. *Human Performance, 22* (2), 128-142.

Mahajan, A., Bishop, J.W., & Scott, D. (2012). Does trust in top management mediate top management communication, employee involvement and organizational commitment relationships*? Journal of Managerial Issues*, *24* (2), 173-190.

Mahony, D.M., Klimchak, M., & Morrell, D.L. (2012). The portability of career-long work experience: Propensity to trust as a substitute for valuable work experience. *Career Development International, 17* (7), 606-625.

Mallin, M.L., O'Donnell, E., & Hu, M.Y. (2009). The role of uncertainty and sales control in the development of sales manager trust. *Journal of Business & Industrial Marketing, 25* (1), 30-42.

Mayer, R.C., & Gavin, M.B. (2005). Trust in management and performance: Who minds the shop while the employees watch the boss? *Academy of Management Journal, 48* (5), 874-888.

Mealy, M., Stephan, W.G., Mhaka-Mutepfa, M., & Alvarado-Sanchez, L. (2015). Interpersonal Trust in Ecuador, the United States, and Zimbabwe. *Cross-Cultural Research, 49* (4), 393-421.

Miao, Q., Newman, A., & Huang, X. (2014). The impact of participative leadership on job performance and organizational citizenship behavior: Distinguishing between the mediating effects of affective and cognitive trust. *The International Journal of Human Resource Management, 25* (20), 2796-2810.

Mitchell, R.M., Ripley, J., Adams, C., & Raju, D. (2011). Trust an essential ingredient in collaborative decision making. *Journal of School Public Relations, 32* (2), 145-170.

Morrow Jr, J.L., Hansen, M.H., & Pearson, A.W. (2004). The cognitive and affective antecedents of general trust within cooperative organizations. *Journal of Managerial Issues*, *16* (1), 48-64.

Moshtari, M. (2016). Inter‐organizational fit, relationship management capability, and collaborative performance within a humanitarian setting. *Production and Operations Management, 25* (9), 1542-1557.

Muethel, M., Siebdrat, F., & Hoegl, M. (2012). When do we really need interpersonal trust in globally dispersed new product development teams? *R&D Management, 42* (1), 31-46.

Mushonga, S.M., Thiagarajan, P., & Torrance, C.G. (2014). Fairness in the workplace: The mediating role of trust in the relationship between supervisory justice and work outcomes. *SAM Advanced Management Journal, 79* (3), 17-25.

Nambudiri, R. (2012). Propensity to trust and organizational commitment: a study in the Indian pharmaceutical sector. *The International Journal of Human Resource Management, 23* (5), 977-986.

Neves, P., & Caetano, A. (2009). Commitment to change: Contributions to trust in the supervisor and work outcomes. *Group & Organization Management, 34* (6), 623-644.

Newman, A., Rose, P. S., & Teo, S.T. (2016). The role of participative leadership and trust‐based mechanisms in eliciting intern performance: Evidence from China. *Human Resource Management, 55* (1), 53-67.

Nielsen, B.B. (2007). Determining international strategic alliance performance: A multidimensional approach. *International Business Review, 16* (3), 337-361.

Nikandrou, I., Papalexandris, N., & Bourantas, D. (2000). Gaining employee trust after acquisition: implications for managerial action. *Employee Relations, 22* (4), 334-355.

Northouse, P.G. (1979). Interpersonal trust and empathy in nurse-nurse relationships*. Nursing Research, 28* (6), 365-368.

Oh, Y., & Park, J. (2011). New link between administrative reforms and job attitude: The role of interpersonal trust in peers as a mediator on organizational commitment. *International Review of Public Administration, 16* (3), 65-87.

Oliveira, J.M., & Scherbaum, C. (2016). Effects of Activating Team Diversity Dimensions on Member Perceptions of Conflict, Trust, and Respect. *The New School Psychology Bulletin, 13* (1), 21-37.

Olson, B. J., Parayitam, S., & Bao, Y. (2007). Strategic decision making: The effects of cognitive diversity, conflict, and trust on decision outcomes. *Journal of Management, 33* (2), 196-222.

Palanski, M.E., & Yammarino, F.J. (2011). Impact of behavioral integrity on follower job performance: A three-study examination. *The Leadership Quarterly, 22* (4), 765-786.

Palanski, M.E., Kahai, S.S., & Yammarino, F.J. (2011). Team virtues and performance: An examination of transparency, behavioral integrity, and trust. *Journal of Business Ethics, 99* (2), 201-216.

Panchamia, J. (2013). A study of OCTAPACE culture in civil hospital, Gandhinagar. IUP *Journal of Organizational Behavior, 12* (2), 43-50.

Pappas, J.M., & Flaherty, K.E. (2008). The effect of trust on customer contact personnel strategic behavior and sales performance in a service environment. *Journal of Business Research, 61* (9), 894-902.

Parayitam, S., & Dooley, R.S. (2009). The interplay between cognitive-and affective conflict and cognition-and affect-based trust in influencing decision outcomes. *Journal of Business Research, 62* (8), 789-796.

Parayitam, S., Olson, B. J., & Bao, Y. (2010). Task conflict, relationship conflict and agreement-seeking behavior in Chinese top management teams. *International Journal of Conflict Management, 21* (1), 94-116.

Parayitam, S., & Papenhausen, C. (2016). Agreement-seeking behavior, trust, and cognitive diversity in strategic decision-making teams: Process conflict as a moderator*. Journal of Advances in Management Research, 13* (3), 292-315.

Park, H., Gowan, M., & Dai Hwang, S. (2002). Impact of national origin and entry mode on trust and organizational commitment. *Multinational Business Review, 10* (2), 52-61.

Park, J.G., & Lee, J. (2014). Knowledge sharing in information systems development projects: Explicating the role of dependence and trust. *International Journal of Project Management, 32* (1), 153-165.

Parker, S.K., Williams, H.M., & Turner, N. (2006). Modeling the antecedents of proactive behavior at work. *Journal of Applied Psychology, 91* (3), 636-652.

Paulson, G.D., & Naquin, C.E. (2004). Establishing trust via technology: Long distance practices and pitfalls. *International Negotiation, 9* (2), 229-244.

Pearce, J.L., Branyiczki, I., & Bigley, G.A. (2000). Insufficient bureaucracy: Trust and commitment in particularistic organizations*. Organization Science, 11* (2), 148-162.

Peralta, C.F., & Saldanha, M.F., (2014). Knowledge-centered culture and knowledge sharing: the moderator role of trust propensity. *Journal of Knowledge Management, 18* (3), 538-550.

Peters, L., & Karren, R.J. (2009). An examination of the roles of trust and functional diversity on virtual team performance ratings. *Group & Organization Management, 34* (4), 479-504.

Peterson, R.S., & Behfar, K.J. (2003). The dynamic relationship between performance feedback, trust, and conflict in groups: A longitudinal study. *Organizational Behavior and Human Decision Processes, 92* (1), 102-112.

Podsakoff, P.M., MacKenzie, S.B., Moorman, R.H., & Fetter, R. (1990). Transformational leader behaviors and their effects on followers' trust in leader, satisfaction, and organizational citizenship behaviors. *The Leadership Quarterly,* *1* (2), 107-142.

Politis, J.D. (2003). The connection between trust and knowledge management: what are its implications for team performance. *Journal of Knowledge Management, 7* (5), 55-66.

Poon, J.M. (2013). Effects of benevolence, integrity, and ability on trust-in-supervisor. *Employee Relations, 35* (4), 396-407.

Poon, J.M., Salleh, A. H.M., & Senik, Z.C. (2007). Propensity to trust as a moderator of the relationship between perceived organizational support and job satisfaction. *International Journal of Organization Theory and Behavior, 10* (3), 350-365.

Porter, T.W., & Lilly, B.S. (1996). The effects of conflict, trust, and task commitment on project team performance. International *Journal of Conflict Management, 7* (4), 361-376.

Praxmarer-Carus, S. (2014). Why the proposal of a complex contract may harm or foster a partner's trust. *Journal of Business Research, 67* (7), 1421-1429.

Qiu, T., & Peschek, B.S. (2012). The effect of interpersonal counterproductive workplace behaviors on the performance of new product development teams. *American Journal of Management, 12* (1), 21-33.

Radnitz, S., Wheatley, J., & Zürcher, C. (2009). The origins of social capital: Evidence from a survey of post-soviet central Asia. *Comparative Political Studies, 42* (6), 707-732.

Ramos, T., Oliveira, M., Santos, A.S., Garcia-Marques, L., & Carneiro, P. (2016). Evaluating young and old faces on social dimensions: Trustworthiness and dominance. *Psicológica, 37* (2), 169-185.

Rao, A.N. (2015). Trust and Team Performance: Assessing the Moderating Role of Risk in Global Outsourcing Teams. *International Management Review, 11* (1), 5-16.

Rao, G.S. (1975). Interpersonal Trust and Its Correlates as Perceived by Superiors and Subordinates. Indian *Journal of Industrial Relations*, *10* (3), 359-369.

Rast III, D.E., Hogg, M.A., & Giessner, S.R. (2016). Who trusts charismatic leaders who champion change? The role of group identification, membership centrality, and self-uncertainty. *Group Dynamics: Theory, Research, and Practice, 20* (4), 259.

Ratan, R.A., Chung, J.E., Shen, C., Williams, D., & Poole, M.S. (2010). Schmoozing and smiting: Trust, social institutions, and communication patterns in an MMOG. *Journal of Computer‐Mediated Communication, 16* (1), 93-114.

Rau, D. (2005). The influence of relationship conflict and trust on the transactive memory: Performance relation in top management teams. *Small Group Research, 36* (6), 746-771.

Rezlescu, C., Duchaine, B., Olivola, C.Y., & Chater, N. (2012). Unfakeable facial configurations affect strategic choices in trust games with or without information about past behavior. *PloS One, 7* (3), e34293.

Rico, R., Alcover, C.M., Sánchez-Manzanares, M., & Gil, F. (2009). The joint relationships of communication behaviors and task interdependence on trust building and change in virtual project teams. *Social Science Information, 48* (2), 229-255.

Ristig, K. (2009). The impact of perceived organizational support and trustworthiness on trust. *Management Research News, 32* (7), 659-669.

Robert, L.P., Denis, A.R., & Hung, Y.T.C. (2009). Individual swift trust and knowledge-based trust in face-to-face and virtual team members. *Journal of Management Information Systems, 26* (2), 241-279.

Roberts, K.H., & O'Reilly, C.A. (1974). Failures in upward communication in organizations: Three possible culprits. *Academy of Management Journal, 17* (2), 205-215.

Robertson, R., Gockel, C., & Brauner, E. (2012). Trust your teammates or bosses? Differential effects of trust on transactive memory, job satisfaction, and performance. *Employee Relations, 35* (2), 222-242.

Rockmann, K.W., & Northcraft, G.B. (2008). To be or not to be trusted: The influence of media richness on defection and deception. *Organizational Behavior and Human Decision Processes, 107* (2), 106-122.

Rockwell, P., & Hubbard, A.E. (1999). The effect of attorneys ‘nonverbal communication on perceived credibility. *The Journal of Credibility Assessment and Witness Psychology, 2* (1), 1-13.

Romeike, P.D., Nienaber, A.M., & Schewe, G. (2016). How differences in perceptions of own and team performance impact trust and job satisfaction in virtual teams. *Human Performance, 29* (4), 291-309.

Roussin, C.J., & Webber, S.S. (2012). Impact of organizational identification and psychological safety on initial perceptions of coworker trustworthiness. *Journal of Business and Psychology, 27* (3), 317-329.

Saccardi, T.A., & Banai, M. (1996). The effects of hospital executives' personality traits on their perceptions and trust. *Journal of Healthcare Management, 41* (2), 197-209.

Saud Khan, M.J. Breitenecker, R., & Schwarz, E. (2014). Entrepreneurial team locus of control: diversity and trust. *Management Decision, 52* (6), 1057-1081.

Saygı, Ö., Greer, L.L., van Kleef, G.A., & De Dreu, C.K. (2014). Competitive representative negotiations worsen intergroup relations. *Group Processes & Intergroup Relations, 17* (2), 143-160.

Scarbrough, J.E. (2013). Student-faculty trust and student success in pre-licensure baccalaureate nurse education. *Nurse Education Today, 33* (8), 919-924.

Schaubroeck, J., Lam, S.S., & Peng, A.C. (2011). Cognition-based and affect-based trust as mediators of leader behavior influences on team performance. *Journal of Applied Psychology, 96* (4), 863.

Serva, M.A., Fuller, M.A., & Mayer, R.C. (2005). The reciprocal nature of trust: A longitudinal study of interacting teams. *Journal of Organizational Behavior, 26* (6), 625-648.

Shamir, B., & Lapidot, Y. (2003). Trust in organizational superiors: Systemic and collective considerations. *Organization Studies, 24* (3), 463-491.

Sheng-Yi, W.U., Shih-Ting, W.A.N.G., Liu, E.Z.F., Da-Chain, H.U., & Hwang, W.Y. (2012). The influences of social self-efficacy on social trust and social capital-A case study of Facebook. *TOJET: The Turkish Online Journal of Educational Technology, 11* (2), 246-254.

Sholihin, M., & Pike, R. (2010). Organizational commitment in the police service: exploring the effects of performance measures, procedural justice and interpersonal trust. *Financial Accountability & Management, 26* (4), 392-421.

Sholihin, M., Pike, R., Mangena, M., & Li, J. (2011). Goal-setting participation and goal commitment: Examining the mediating roles of procedural fairness and interpersonal trust in a UK financial services organization. *The British Accounting Review, 43* (2), 135-146.

Singh, U., & Srivastava, K. B. (2009). Interpersonal trust and organizational citizenship behavior. *Psychological Studies, 54* (1), 65-76.

Small, E.E., & Rentsch, J.R. (2010). Shared leadership in teams: A matter of distribution. *Journal of Personnel Psychology, 9* (4), 203-211.

Smith, F.G., Jones, B.C., Little, A.C., DeBruine, L.M., Welling, L.L., Vukovic, J., & Conway, C.A. (2009). Hormonal contraceptive use and perceptions of trust modulate the effect of relationship context on women's preferences for sexual dimorphism in male face shape. *Journal of Evolutionary Psychology, 7* (3), 195-210.

Solomonson, W.L. (2012). Trust and the client–consultant relationship. *Performance Improvement Quarterly, 25* (3), 53-80.

Son, S., & Kim, D.Y. (2016). The role of perceived management support and trust in mentors on protégés’ organizational citizenship behavior. *Asia Pacific Journal of Human Resources, 54* (4), 481-497.

Song, F., Cadsby, C.B., & Bi, Y. (2012). Trust, reciprocity, and guanxi in China: An experimental investigation. *Management and Organization Review, 8* (2), 397-421.

Spreitzer, G.M., & Mishra, A.K. (1999). Giving up control without losing control: Trust and its substitutes’ effects on managers’ involving employees in decision making. *Group & Organization Management, 24* (2), 155-187.

Stiff, C. (2008). Are they bothered? How the opportunity to damage a partner's reputation influences giving behavior in a trust game. *The Journal of Social Psychology, 148* (5), 609-630.

Su, C.J. (2010). An examination of the usage and impact of upward influence tactics by workers in the hospitality sector of Taiwan: expanding the framework of Rao, Schmidt, and Murray (1995). Canadian *Journal of Administrative Sciences/Revue Canadienne des Sciences de l'Administration, 27* (4), 306-319.

Sue-Chan, C., Au, A.K., & Hackett, R.D. (2012). Trust as a mediator of the relationship between leader/member behavior and leader-member-exchange quality. *Journal of World Business, 47* (3), 459-468.

Swol, L.M., & Sniezek, J.A. (2005). Factors affecting the acceptance of expert advice. *British Journal of Social Psychology, 44* (3), 443-461.

Tam, T., Hewstone, M., Kenworthy, J., & Cairns, E. (2009). Intergroup trust in Northern Ireland. Personality and Social Psychology Bulletin, 35(1), 45-59.

Tan, H.H., & Lim, A. K. (2009). Trust in coworkers and trust in organizations. *The Journal of Psychology, 143* (1), 45-66.

Thacker, R A. (1999). Perceptions of trust, upward influence tactics, and performance ratings. *Perceptual & Motor Skills, 88* (3_suppl), 1059-1070.

Thielmann, I., & Hilbig, B.E. (2014). Trust in me, trust in you: A social projection account of the link between personality, cooperativeness, and trustworthiness expectations. *Journal of Research in Personality, 50*, 61-65.

Tschannen-Moran, M., & Gareis, C.R. (2015). Faculty trust in the principal: An essential ingredient in high-performing schools. *Journal of Educational Administration, 53* (1), 66-92.

Tseng, H.W., & Yeh, H.T. (2013). Team members' perceptions of online teamwork learning experiences and building teamwork trust: A qualitative study. *Computers & Education, 63*, 1-9.

Tyagi, P.K. (1985). Relative importance of key job dimensions and leadership behaviors in motivating salesperson work performance. *The Journal of Marketing*, *49* (3), 76-86.

Tzafrir, S.S. (2005). The relationship between trust, HRM practices and firm performance. The International *Journal of Human Resource Management, 16* (9), 1600-1622.

Tzafrir, S.S., & Eitam-Meilik, M. (2005). The impact of downsizing on trust and employee practices in high tech firms: A longitudinal analysis. *The Journal of High Technology Management Research, 16* (2), 193-207.

Tzafrir, S.S., late Gedaliahu, H., Harel, Baruch, Y., & Dolan, S.L. (2004). The consequences of emerging HRM practices for employees' trust in their managers. *Personnel Review, 33* (6), 628-647.

Ugwu, L.I., Enwereuzor, I.K., & Orji, E.U. (2016). Is trust in leadership a mediator between transformational leadership and in-role performance among small-scale factory workers? *Review of Managerial Science, 10* (4), 629-648.

Vakola, M. (2014). What's in there for me? Individual readiness to change and the perceived impact of organizational change. *Leadership & Organization Development Journal, 35* (3), 195-209.

Van Dam, K., Oreg, S., & Schyns, B. (2008). Daily work contexts and resistance to organisational change: The role of leader–member exchange, development climate, and change process characteristics. *Applied Psychology, 57* (2), 313-334.

Van Dyne, L., Vandewalle, D., Kostova, T., Latham, M.E., & Cummings, L.L. (2000). Collectivism, propensity to trust and self‐esteem as predictors of organizational citizenship in a non‐work setting. *Journal of Organizational Behavior*, *21* (1), 3-23.

Van Maele, D., & Van Houtte, M. (2009). Faculty trust and organizational school characteristics: An exploration across secondary schools in Flanders. *Educational Administration Quarterly, 45* (4), 556-589.

Van Rinsum, M., & Verbeeten, F.H. (2012). The impact of subjectivity in performance evaluation practices on public sector managers’ motivation. *Accounting and Business Research, 42* (4), 377-396.

Van Den Berg, P.T., & Van Der Velde, M.E. (2005). Relationships of functional flexibility with individual and work factors. *Journal of Business and Psychology, 20* (1), 111-129.

van der Werff, L., & Buckley, F. (2017). Getting to know you: A longitudinal examination of trust cues and trust development during socialization. *Journal of Management, 43* (3), 742-770.

Voci, A. (2006). The link between identification and in‐group favouritism: Effects of threat to social identity and trust‐related emotions. *British Journal of Social Psychology, 45* (2), 265-284.

Vukovic, J., Jones, B.C., Feinberg, D.R., DeBruine, L.M., Smith, F.G., Welling, L.L., & Little, A.C. (2011). Variation in perceptions of physical dominance and trustworthiness predicts individual differences in the effect of relationship context on women's preferences for masculine pitch in men's voices. *British Journal of Psychology, 102* (1), 37-48.

Wai On, L., Liang, X., Priem, R., & Shaffer, M. (2013). Top management team trust, behavioral integration, and the performance of international joint ventures. *Journal of Asia Business Studies, 7* (2), 99-122.

Walker, B.A., & Robinson, R. (1979). Utilizing dimensions of the Rotter interpersonal trust scale in investigations of trust: validation of suggested methods. *Psychological Reports, 44* (2), 423-429.

Walsh, G., Gouthier, M., Gremler, D.D., & Brach, S. (2012). What the eye does not see, the mind cannot reject: Can call center location explain differences in customer evaluations? *International Business Review, 21* (5), 957-967.

Walther, J.B., & Bunz, U. (2005). The rules of virtual groups: Trust, liking, and performance in computer‐mediated communication. *Journal of Communication, 55* (4), 828-846.

Wang, C.S., & Leung, A.K.Y. (2010). The cultural dynamics of rewarding honesty and punishing deception. *Personality and Social Psychology Bulletin, 36 (*11), 1529-1542.

Wang, D.S., & Hsieh, C.C. (2013). The effect of authentic leadership on employee trust and employee engagement. *Social Behavior and Personality: An International Journal, 41* (4), 613-624.

Wang, Q., & Bowen, S.P. (2014). The limits of beauty: Effects of physician attractiveness and biological sex on patient trust, satisfaction, and disclosure. *Communication Research Reports, 31* (1), 72-81.

Wang, X., & Li, Y. (2017). How trust and need satisfaction motivate producing user-generated content. *Journal of Computer Information Systems, 57* (1), 49-57.

Wang, Y. (2003). Trust and decision-making styles in Chinese township-village enterprises. *Journal of Managerial Psychology, 18* (6), 541-556.

Wang, C., & Bird, J J. (2011). Multi-level modeling of principal authenticity and teacher trust and engagement. *Academy of Educational Leadership Journal, 15* (4), 125-148.

Wasti, S.A., Tan, H.H., Brower, H.H., & Önder, Ç. (2007). Cross-cultural measurement of supervisor trustworthiness: An assessment of measurement invariance across three cultures. *The Leadership Quarterly, 18* (5), 477-489.

Webber, S.S. (2008). Blending service provider–client project teams to achieve client trust: Implications for project team trust, cohesion, and performance. *Project Management Journal, 39* (2), 72-81.

Webber, S.S., & Klimoski, R J. (2004). Client–project manager engagements, trust, and loyalty. *Journal of Organizational Behavior, 25* (8), 997-1013.

Webber, S.S., Payne, S.C., & Taylor, A.B. (2012). Personality and trust fosters service quality. *Journal of Business and Psychology, 27* (2), 193-203.

Webber, S.S. (2008). Development of cognitive and affective trust in teams: A longitudinal study. *Small Group Research, 39* (6), 746-769.

Wells, C.V., & Kipnis, D. (2001). Trust, dependency, and control in the contemporary organization. *Journal of Business and Psychology, 15 (*4), 593-603.

Winkielman, P., Olszanowski, M., & Gola, M. (2015). Faces in-between: Evaluations reflect the interplay of facial features and task-dependent fluency. *Emotion, 15* (2), 232-243.

Wöhrle, J., van Oudenhoven, J.P., Otten, S., & van der Zee, K.I. (2015). Personality characteristics and workplace trust of majority and minority employees in the Netherlands. *European Journal of Work and Organizational Psychology, 24* (2), 161-177.

Wong, C.A., & Cummings, G.G. (2009). The influence of authentic leadership behaviors on trust and work outcomes of health care staff. *Journal of Leadership Studies, 3* (2), 6-23.

Wong, Y.T., Wong, C.S., & Ngo, H.Y. (2002). Loyalty to supervisor and trust in supervisor of workers in Chinese joint ventures: A test of two competing models. *International Journal of Human Resource Management, 13* (6), 883-900.

Workman, M. (2009). A field study of corporate employee monitoring: Attitudes, absenteeism, and the moderating influences of procedural justice perceptions. *Information and Organization, 19* (4), 218-232.

Wu, M., Huang, X., & Chan, S.C. (2012). The influencing mechanisms of paternalistic leadership in Mainland China. *Asia Pacific Business Review, 18* (4), 631-648.

Wu, M., Huang, X., Li, C., & Liu, W. (2012). Perceived interactional justice and trust‐in‐supervisor as mediators for paternalistic leadership. *Management and Organization Review, 8* (1), 97-121.

Wu, S.Y., Wang, S.T., Liu, F., Hu, D.C., & Hwang, W.Y. (2012). The Influences of Social Self-Efficacy on Social Trust and Social Capital--A Case Study of Facebook. *Turkish Online Journal of Educational Technology-TOJET*, *11* (2), 246-254.

Wu, W.L., & Lee, Y.C. (2016). How to make a knowledge-sharing group: a group social capital perspective. *Personnel Review, 45 (*3), 523-538.

Yıldız, I.G., & Şimşek, Ö.F. (2016). Different pathways from transformational leadership to job satisfaction. *Nonprofit Management and Leadership, 27* (1), 59-77.

Yakovleva, M., Reilly, R.R., & Werko, R. (2010). Why do we trust? Moving beyond individual to dyadic perceptions. *Journal of Applied Psychology, 95* (1), 79-91.

Yang, J., Mossholder, K.W., & Peng, T K. (2009). Supervisory procedural justice effects: The mediating roles of cognitive and affective trust*. The Leadership Quarterly, 20* (2), 143-154.

Yen, Y.F., Tseng, J.F., & Wang, H.K. (2014). Exploring the mediating role of trust on the relationship between guanxi and knowledge sharing: a social network perspective. *Asia Pacific Journal of Human Resources, 52* (2), 173-192.

Yoon, M.H., & Suh, J. (2003). Organizational citizenship behaviors and service quality as external effectiveness of contact employees. *Journal of Business Research, 56* (8), 597-611.

Zaheer, A., McEvily, B., & Perrone, V. (1998). Does trust matter? Exploring the effects of interorganizational and interpersonal trust on performance. *Organization Science, 9* (2), 141-159.

Zapata, C.P., Olsen, J.E., & Martins, L.L. (2013). Social exchange from the supervisor’s perspective: Employee trustworthiness as a predictor of interpersonal and informational justice. *Organizational Behavior and Human Decision Processes, 121* (1), 1-12.

Zaqout, F., & Abbas, M. (2012). Towards a model for understanding the influence of the factors that stimulate university students' engagement and performance in knowledge sharing. *Library Review, 61* (5), 345-361.

Zeffane, R. (2015). Trust, personality, risk taking and entrepreneurship: Exploring gender differences among nascent and actual entrepreneurs in the United Arab Emirates. *World Journal of Entrepreneurship, Management and Sustainable Development, 11* (3), 191-209.

Zell, D., McGrath, C., & Vance, C. M. (2014). Examining the interaction of extroversion and network structure in the formation of effective informal support networks. *Journal of Behavioral and Applied Management, 15* (2), 59-81.

Zhang, J.D., Liu, L.A., & Liu, W. (2015). Trust and deception in negotiation: Culturally divergent effects. *Management and Organization Review*, *11*(1), 123-144.

Zhang, L., Cheng, J., & Wang, D. (2015). The influence of informal governance mechanisms on knowledge integration within cross-functional project teams: a social capital perspective. *Knowledge Management Research & Practice, 13* (4), 508-516.

Zhang, L., & Morand, D. (2014). The linkage between status-leveling symbols and work attitudes. *Journal of Managerial Psychology, 29* (8), 973-993.

Zhang, X., & Zhou, J. (2014). Empowering leadership, uncertainty avoidance, trust, and employee creativity: Interaction effects and a mediating mechanism. *Organizational Behavior and Human Decision Processes, 124* (2), 150-164.

Zhao, N., & Zhang, J. (2016). Gender differences in trusting strangers: Role of the target's gender. *PsyCh Journal, 5* (2), 83-91.

Zheng, X., Yin, H., Liu, Y., & Ke, Z. (2016). Effects of leadership practices on professional learning communities: The mediating role of trust in colleagues. *Asia Pacific Education Review, 17* (3), 521-532.

Zheng, Y. (2012). Unlocking founding team prior shared experience: A transactive memory system perspective. *Journal of Business Venturing, 27* (5), 577-591.

Zhu, W., Newman, A., Miao, Q., & Hooke, A. (2013). Revisiting the mediating role of trust in transformational leadership effects: Do different types of trust make a difference? *The Leadership Quarterly, 24* (1), 94-105.

Zhu, Y., & Akhtar, S. (2014). The mediating effects of cognition-based trust and affect-based trust in transformational leadership's dual processes: evidence from China. *The International Journal of Human Resource Management, 25* (20), 2755-2771.

Zoghbi-Manrique-de-Lara, P., & Ting-Ding, J.M. (2016). The influence of corporate culture and workplace relationship quality on the outsourcing success in hotel firms. *International Journal of Hospitality Management, 56*, 66-77.

Zornoza, A., Orengo, V., & Peñarroja, V. (2009). Relational capital in virtual teams: the role played by trust. *Social Science Information, 48* (2), 257-281.

Zwikael, O., & Smyrk, J. (2015). Project governance: Balancing control and trust in dealing with risk. *International Journal of Project Management, 33* (4), 852-862.
